# Phase I Randomised Clinical Trial of an HIV-1_CN54_, Clade C, Trimeric Envelope Vaccine Candidate Delivered Vaginally

**DOI:** 10.1371/journal.pone.0025165

**Published:** 2011-09-30

**Authors:** David J. Lewis, Carol A. Fraser, Abdel N. Mahmoud, Rebecca C. Wiggins, Maria Woodrow, Alethea Cope, Chun Cai, Rafaela Giemza, Simon A. Jeffs, Maria Manoussaka, Tom Cole, Martin P. Cranage, Robin J. Shattock, Charles J. Lacey

**Affiliations:** 1 Centre for Infection & Immunity, Division of Clinical Sciences, St George's, University of London, United Kingdom; 2 Hull York Medical School & Centre for Immunology and Infection, University of York, United Kingdom; 3 Jefferiss Trust Research Laboratories, Imperial College, London, United Kingdom; New York Blood Center, United States of America

## Abstract

We conducted a phase 1 double-blind randomised controlled trial (RCT) of a HIV-1 envelope protein (CN54 gp140) candidate vaccine delivered vaginally to assess immunogenicity and safety. It was hypothesised that repeated delivery of gp140 may facilitate antigen uptake and presentation at this mucosal surface. Twenty two healthy female volunteers aged 18–45 years were entered into the trial, the first receiving open-label active product. Subsequently, 16 women were randomised to receive 9 doses of 100 µg of gp140 in 3 ml of a Carbopol 974P based gel, 5 were randomised to placebo solution in the same gel, delivered vaginally via an applicator. Participants delivered the vaccine three times a week over three weeks during one menstrual cycle, and were followed up for two further months. There were no serious adverse events, and the vaccine was well tolerated. No sustained systemic or local IgG, IgA, or T cell responses to the gp140 were detected following vaginal immunisations. Repeated vaginal immunisation with a HIV-1 envelope protein alone formulated in Carbopol gel was safe, but did not induce local or systemic immune responses in healthy women.

**Trial Registration:**

ClinicalTrials.gov NCT00637962

## Introduction

The need to develop an effective HIV vaccine remains imperative. There is growing evidence that an effective vaccine against HIV-1 will need to induce broadly protective immune responses at the virus portal of entry which can be recalled at sufficient magnitude shortly after *in vivo* infectious challenge [1], [2].

Our specific interest is to investigate vaccine strategies which attempt to invoke persistent genital tract as well as systemic immune responses that might be sufficient to block female receptive vaginal HIV-1 transmission. Data from macaque SIV-challenge models suggests that HIV is able to traverse the vaginal mucosa within a few hours, and establish infection within 24 hours [3]. Although systemic immunisation is able to block sexual transmission in women of some pathogens (e.g. HPV) [4], it is not clear whether this approach will be effective against HIV, which directly targets and subverts the immune system. Therefore our goal is to investigate novel vaccine strategies that might drive maximal anti-HIV-1 female genital tract mucosal immune responses.

Vaginal vaccination has been explored in women and female non-human primates in a number of systems [5]–[10]. Although some degree of response was reported in all of these experiments, the antigens were usually co-administered with a local adjuvant, such as recombinant cholera toxin B [7]–[10]. These studies demonstrated that the efficiency of vaginal immunisation was influenced by the menstrual cycle [9], [10]. To circumvent this variable we hypothesised that repeated exposure to antigen throughout the menstrual cycle may be most likely to generate an immune response. Furthermore, we used HIV-1_CN54_ clade C recombinant trimeric envelope (gp140), which maintains CD4-binding function, and is to some extent expected to emulate natural exposure to HIV-1 envelope protein. We chose to avoid the use of mucosal adjuvants in the vagina because of the inherent dangers of up-regulating target cells and HIV transmission [11]. For the first human study in our programme, we therefore determined whether nine vaginal immunisations with an HIV-1 envelope protein alone, administered throughout one menstrual cycle, could induce genital tract and systemic immunity. We had performed a series of toxicological, immunological, and formulation studies in rabbits prior to the human trial, and were encouraged by the results showing that formulations of the CN54 gp140 trimer were well tolerated and induced both serum and mucosal antibody responses after a single cycle of vaginal immunisation [12], [13].

## Methods

### 1.1 RCT

This was a randomised, placebo-controlled, double-blind phase 1 clinical trial. The protocol for this trial and supporting CONSORT checklist are available as supporting information; see Checklist S1 and Protocol S1. The trial was conducted at two centres, the Vaccine Institute, St George's, University of London, and the Department of GU Medicine, York Hospital, York, UK. We first gained regulatory, ethical, and governance approvals. All volunteers gave fully informed written consent, and the trial was conducted according to the UK Clinical Trials Regulations and Good Clinical Practice guidelines.

### 1.2 Subjects, randomisation and interventions

Subjects were healthy female volunteers aged 18–45 years. Potential participants were screened for medical abnormalities, HIV, HBV, HCV, sexually transmitted infections and gynaecologic disorders, and there was a range of other inclusion and exclusion criteria. All women had to use condoms without a spermicidal agent, and in addition use either combined oral contraception, a diaphragm, or be sterilised. There was a total of 16 study visits over 4 menstrual cycles. The randomisation was computer generated by an independent statistician using a 2∶1 ratio of active: placebo and a target population of 30. This was provided to Polymun Scientific who prepared blinded individual subject doses. Corresponding IMP supplies were used sequentially and provided to the sites in blocks of five. The sample size was chosen based on expected side effects. The first subject was given open label active product as a safety precaution. The vaginal vaccines were prepared by bedside mixing of the carrier gel plus the blinded study products [eitherCN54 gp140 (see below), or placebo buffer] and administered by the volunteers at clinic visits nine times (3 times a week for 3 weeks) during a single menstrual cycle following randomisation. Immunisations were performed by insertion of 3 ml of active or placebo gel into the vagina. Subjects were followed up for 8 weeks after the last immunisation. We collected fractionated sera and peripheral blood mononuclear cells (PBMC), plus endocervical and vaginal mucosal secretions using Weck-Cel™ surgical spears (Medtronic™).

### 1.3 Primary and secondary outcomes

The primary outcomes were safety variables as comprised by local cervico-vaginal AEs (epithelial disruption, erythema, or bleeding) and systemic AEs, either by report including diary card, or by clinical examination including colposcopy, and laboratory AEs. Adverse events, both local and systemic, and serious adverse events were recorded and graded according to pre-defined criteria. The secondary outcomes were immunological responses, (i) IgG & IgA responses to CN54 gp140 in vaginal & cervical secretions and serum, (ii) T-cell responses to HIV gp140 peptides in blood, and (iii) T cell responses in cervical mononuclear cells.

### 1.4 Vaccines

We designed, expressed and produced under good manufacturing practice (GMP) a trimeric gp140 from a clade C envelope clone p97CN54, kindly provided by H Wolf & R Wagner, University of Regensburg, Germany. The trimeric gp140 (gp120 plus the external domain of gp41), designated as CN54 gp140, was produced as a recombinant protein in CHO cells, and manufactured to GMP specifications by Polymun Scientific, Vienna, Austria (accession number AF286226). The fidelity of the product was confirmed by mass spectrometry of tryptic fragments by the Medical Biomics Centre at St George's, University of London. An aqueous gel using Carbopol 974P as the matrix-former, and benzyl alcohol as preservative, was designed for vaginal use by Particle Sciences, Bethlehem PA, US. Directly before each immunisation the CN54 gp140 was added to the gel vehicle, the mixture homogenised by multiple passages between two interconnected syringes, and then loaded into a vaginal applicator for administration. The final gel formulation contained 33 µg/ml of gp140. A full battery of toxicology, stability and rabbit vaginal irritation tests were performed with this material with no significant adverse reactions detected.

### 1.5 Genital tract secretions

Mucosal secretions were sampled using pre-weighed Weck-Cel surgical spears placed either in the cervical os or against the vaginal wall for 2 minutes. After reweighing, secretions were eluted from the sponges using a modified version of a previously described method [12]. In brief the spearheads (having absorbed the vaginal secretions) were snipped into the top chamber of a Spin-X tube (Corning) containing 300 µl of sterile filtered extraction buffer [250 mM NaCl, 1× protease inhibitor cocktail set 1 (Calbiochem) in phosphate buffered saline (PBS)]. The Spin-X tubes were centrifuged at 4°C for 15 minutes at 13,000 g. A repeat extraction was performed by adding an additional 300 µl extraction buffer to the top chamber as described above. Then 8 µl of heat inactivated foetal calf serum was added to pooled secretions from each sample site prior to separation into 200 µl aliquots and freezing at −80°C before batch analysis by ELISA as described below.

### 1.6 Cervical cytobrush sampling

Cervical cytobrush samples were collected at screening and weeks 2, 3, 4, 6, and 8 weeks after the last immunisation. A speculum was inserted into the vagina and the cytobrush (Cytobrush®plus, Medscan) was placed in the cervical os and rotated twice through 360°. Cytobrushes were transferred to sterile 15 ml tubes containing 3 ml transport medium, then placed on ice prior to transport to the laboratory for processing and analysis.

### 1.7 Immunological assays

#### 1.7.1 ELISA for antibody responses in serum and mucosal secretions

Systemic IgG & IgA anti-gp140, and cervical and vaginal total IgG & IgA, and anti-gp140 responses were measured by ELISA. Antigen-specific ELISA assays were initially standardised and optimised using a panel of serum samples from 20 HIV negative and 20 HIV positive volunteers. Briefly, 96 well microtitre plates were coated with 50 µl/well CN54 gp140 at 5 µg/ml gp140 in PBS for 1 hour at 37°C. Plates were then washed 4 times in PBST (PBS+0.05% Tween 20) and blocked by adding PBST+10% FCS for 1 h at 37°C. After washing 4 times as described dilutions of serum or mucosal samples were added and incubated at 37°C for 1 hr. Bound specific IgG or IgA responses were detected with either a goat anti-human IgG-HRP (Sigma) or a goat anti-human IgA biotin conjugate (α-chain specific, KPL laboratories) followed by amplification with avidin peroxidise (Sigma). Following developing by the addition of substrate and stop solution, ELISA plates were read at 450 nm on a Versa Max microplate reader (Molecular Devices). Only samples with absorbance reading of ≥0.2 A_450_ (serum and mucosal) for antigen specific IgG and ≥0.4 (serum) ≥0.2 A_450_ (mucosal) for specific IgA were classified as a ‘response detected’, and these were then subjected to endpoint titration, using the same ELISA antigen-specific method described above.

#### 1.7.2. ELISA for total IgG and IgA in mucosal secretions

Total immunoglobulin concentrations in mucosal secretions were measured by sandwich ELISA. Ninety-six well microtitre plates were coated with either goat anti-human IgG capture antibody (γ-chain specific, Sigma) or goat anti-human IgA (α-chain specific, Serotec) capture antibodies at 2 µg/ml. After washing and blocking as described above mucosal secretions were added and bound total IgG or total IgA detected with goat anti-human IgG (Fc specific, Sigma) or IgA (α-specific, Serotec) HRP conjugates and developed as described above. Standard curves were derived using purified human IgG or IgA (Sigma, UK) and concentrations in mucosal secretions calculated by taking into account the dilution factor derived from the weight of the sample/weight of sample +600(µl extraction buffer volume) assuming a density of 1 mg/µl [8].

#### 1.7.3. IFN-γ ELISpot assay

Fractionated PBMC were analysed for interferon-gamma (IFN-γ) release following stimulation with HIV clade C gp140 peptides and controls using an ELISpot kit^pro^ (Mabtech). Cryopreserved PBMC samples were thawed, adjusted to 4×10^6^ cells/ml in R10 complete medium, and rested overnight at 37°C in an atmosphere of 5% C0_2_. Pre-coated human IFN-γ ELISpot plates (Mabtech) were washed and blocked prior to the addition of stimulated PBMC. Viable PBMC were stimulated with either 4 pools of 15mer peptides overlapping by 11 amino acids to cover the entire gp140 (pool 1: amino terminus of gp120 to V1; pool 2: V2 to V3; pool 3: C3 to carboxy terminus of gp120), pool 4: the entire external domain of gp41, a FEC control peptide pool containing 32 peptides comprising human cytomegalovirus, Epstein Barr virus and influenza virus MHC class I restricted epitopes (ARP7099 CFAR, NIBSC) [14] or polyclonal stimulation with PHA-P (as positive controls) all at a concentration of 5 µg/ml or were unstimulated (negative controls) with media alone. PBMC were plated in triplicate wells per stimulus at 2.5×10^5^ cells per well. ELISpot plates were incubated for 16–24 hours at 37°C 5% C0_2_. After washing, bound IFN-γ producing cells were detected with anti-human IFN-γ -ALP labelled detection antibody and BCIP/NBT substrate according to the manufacturer's instructions. Spots were enumerated using an automated ELISpot reader system and software (AID) and expressed as spot forming units (SFU)/10^6^ PBMC.

#### 1.7.4. T cell enumeration in cervical cytobrush samples

Cytobrush samples were processed within 4 hours of collection. The brushes were squeezed with sterile forceps into the transport medium to remove cells prior to centrifugation at 4°C for 15 minutes at 400×g. Cell pellets were re-suspended in 3 ml transport medium and counted by trypan blue exclusion for live cells. If sufficient viable cells were obtained (>10^5^ cells/ml) they were stained for CD3 (APC), CD4 (PECy7) and CD8 (PE) markers (Becton Dickinson). Parallel PBMC samples were used for compensation settings on an FC500 flow cytometer (Beckman Coulter) and the percentages of cells stained recorded.

## Results

### 2.1 Study population

**Figure 1:**
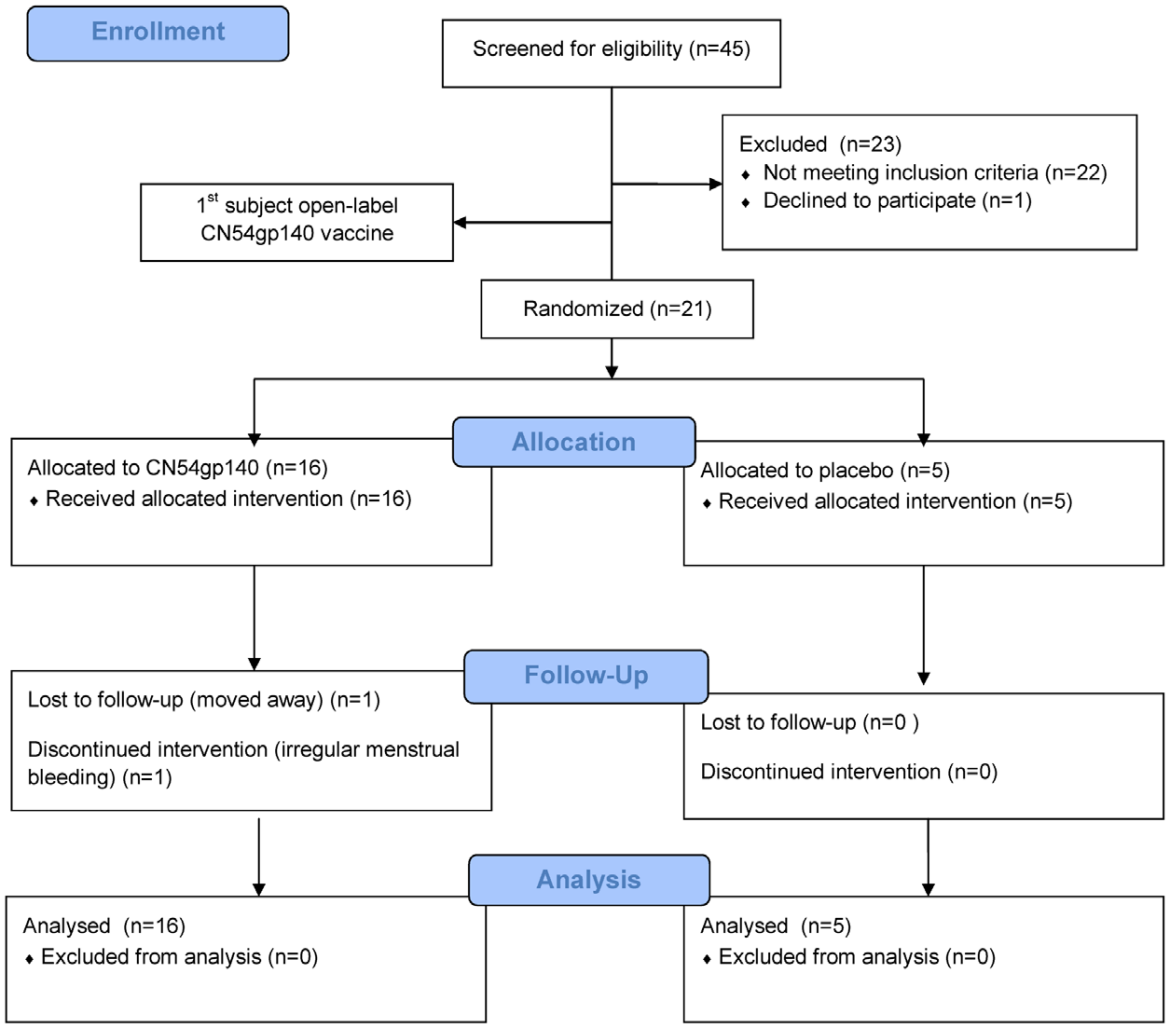
Study flow chart (CONSORT diagram). All subjects screened, enrolled and randomised are detailed. All subjects randomised are included in the safety and immunogenicity populations.

The trial was registered at ClinicalTrials.gov NCT00637962 and approved by the Wandsworth Research Ethics Committee no: 07/Q0803/29. The trial was carried out between October 2007 and October 2008. By October 2008 twenty two women had been screened, were eligible, and had entered and completed the study (Figure 1). No SAEs had been observed and at that point the Data Monitoring Committee advised that the study had fulfilled its objectives and could be terminated. Women were aged 20–43 years, with mean age 26 years, and 85% were of white ethnicity. The first participant was treated with open-label active gp140 immunisations without significant event. Subsequent women were randomised and received blinded product. When the randomisation code was broken it revealed that 16 women received active product and 5 received placebo. The two randomisation groups were well matched for demographic characteristics at entry (Table 1).

**Table 1:** Demographic characteristics of the randomised subjects.

	Placebo	CN54gp140
Number of female subjects	5	16
**Age at screening**		
Mean (SD)	27.0 (6.0)	26.3 (6.1)
Median	24.0	25.0
Range	23–37	20–43
**Weight** (kg)		
Mean (SD)	58.7 (8.4)	62.5 (10.4)
Median	58.0	62.8
Range	48–69	50–85
**Height** (m)		
Mean (SD)	1.65 (0.06)	1.63 (0.05)
Median	1.68	1.64
Range	1.57–1.70	1.55–1.72
**Ethnicity** (n)		
White	5	13
Black	0	2
Asian	0	0
Other	0	1

### 2.2 Safety analyses

There were no serious adverse events reported. All adverse events were enumerated and there were no significant differences in the mean number of events per participant between groups (Table 2). Many of these events were genitourinary symptoms, including vaginal discharge, spotting, vaginal bleeding, vaginal discomfort, etc. One subject received only 8 immunisations and then withdrew consent due to irregular bleeding.

**Table 2:** Summary of adverse events in the randomised subjects.

	Placebo	CN54gp140
	N = 5	N = 16
No. of events (mean)	29 (5.8)	125 (7.8)
No. of local and systemic reactions (mean)	18 (3.6)	72 (4.5)
No. of subjects with any event	5	16
No. of subjects with reproductive system adverse events	4	14
No. of subjects with gastrointestinal system adverse events	2	9
No. of subjects with nervous system adverse events	2	8
No. of subjects with general and administration site adverse events	3	4
No. of subjects with infection adverse events	0	7
Serious adverse events	0	0
Adverse event leading to withdrawal	0	1

### 2.3 Immunogenicity analyses

#### 2.3.1 Serum IgG and IgA gp140 binding antibodies

**Figure 2:**
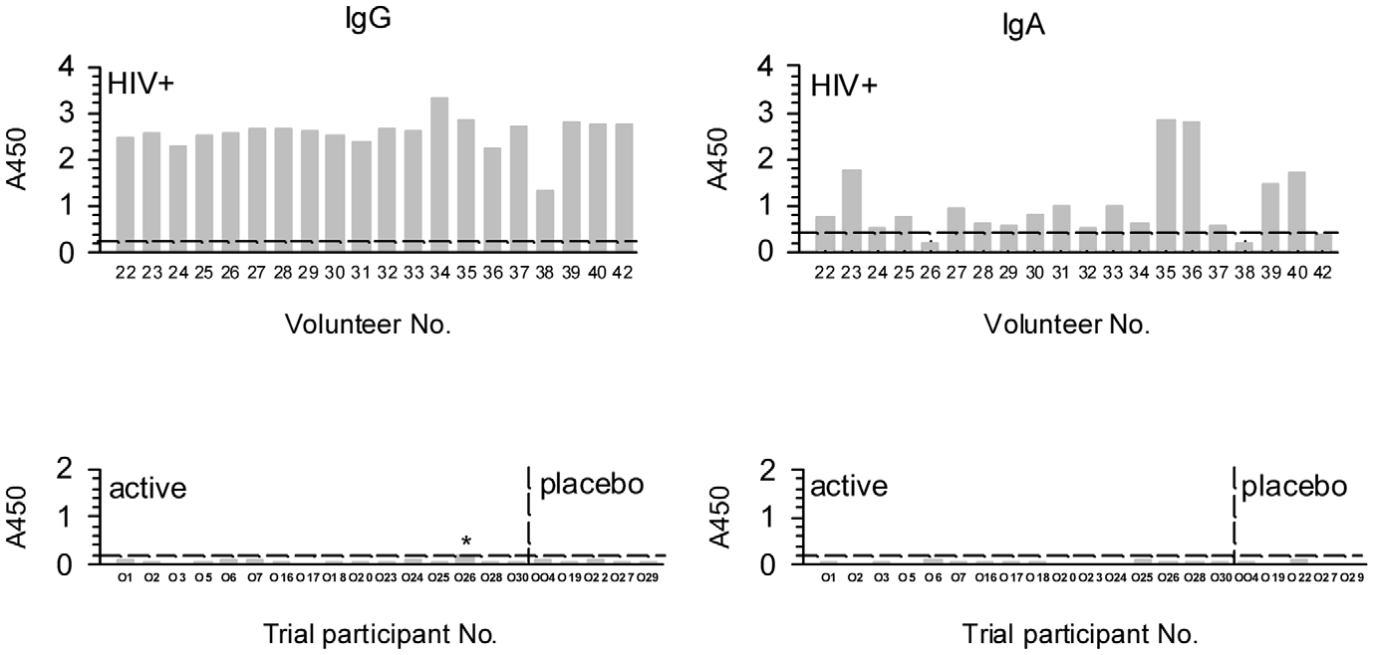
Serum anti-gp140 binding antibodies. Serum samples were screened by gp140-specific ELISA for IgG and IgA antibodies. Assay cut-offs are shown (-------) based on the analysis of sera from 40 uninfected and unvaccinated women. Calibration results with sera from HIV-infected women are shown in the upper panels. Trial participants failed to show positive binding when tested at each visit (lower panels). Representative results are shown for 2 weeks post-last immunisation. * indicates non-specific binding of IgG obtained with sera from trial participant number 026, detected throughout the course of the study period including pre-vaccination.

Initially, a sensitive ELISA for the detection of gp140-specific antibodies was developed. The saturating concentration of recombinant gp140 for solid phase adsorption was determined as 5 µg/ml by checkerboard titration of serum and conjugate dilutions. Although this is a relatively high concentration of antigen it was found to result in reproducible and co-linear serum titration curves even with very low titre sera which were negative in other assay formats (data not shown). For gp140-specific serum IgA detection, the addition of an extra stage of amplification using biotinylated anti-IgA and streptavidin-HRP detector was found to give better discrimination between sera from HIV infected subjects and uninfected individuals (data not shown). The assays were calibrated using sera from 40 HIV-infected and 40 HIV-uninfected women and representative results are shown in Figure 2. 100% specificity and sensitivity was obtained for the detection of anti-gp140 IgG. For IgA antibody 100% specificity and 87.5% sensitivity was obtained at a cut-off of A_450_ 0.3; however, 4 sera from uninfected subjects had values near the cut-off so A_450_ 0.4 was used for screening sera from the clinical trial participants, reducing the sensitivity to 77.5%. Despite the use of these sensitive, standardised assays, anti-gp140 binding antibodies were detected in none of the trial participants (Figure 2). Participant 026 had a relatively high background binding for IgG antibody (at the cut-off) on both pre-vaccination visits but this did not vary during the course of vaccination.

#### 2.3.2 Cervico-vaginal immunoglobulins and anti-gp140 binding antibodies

**Figure 3:**
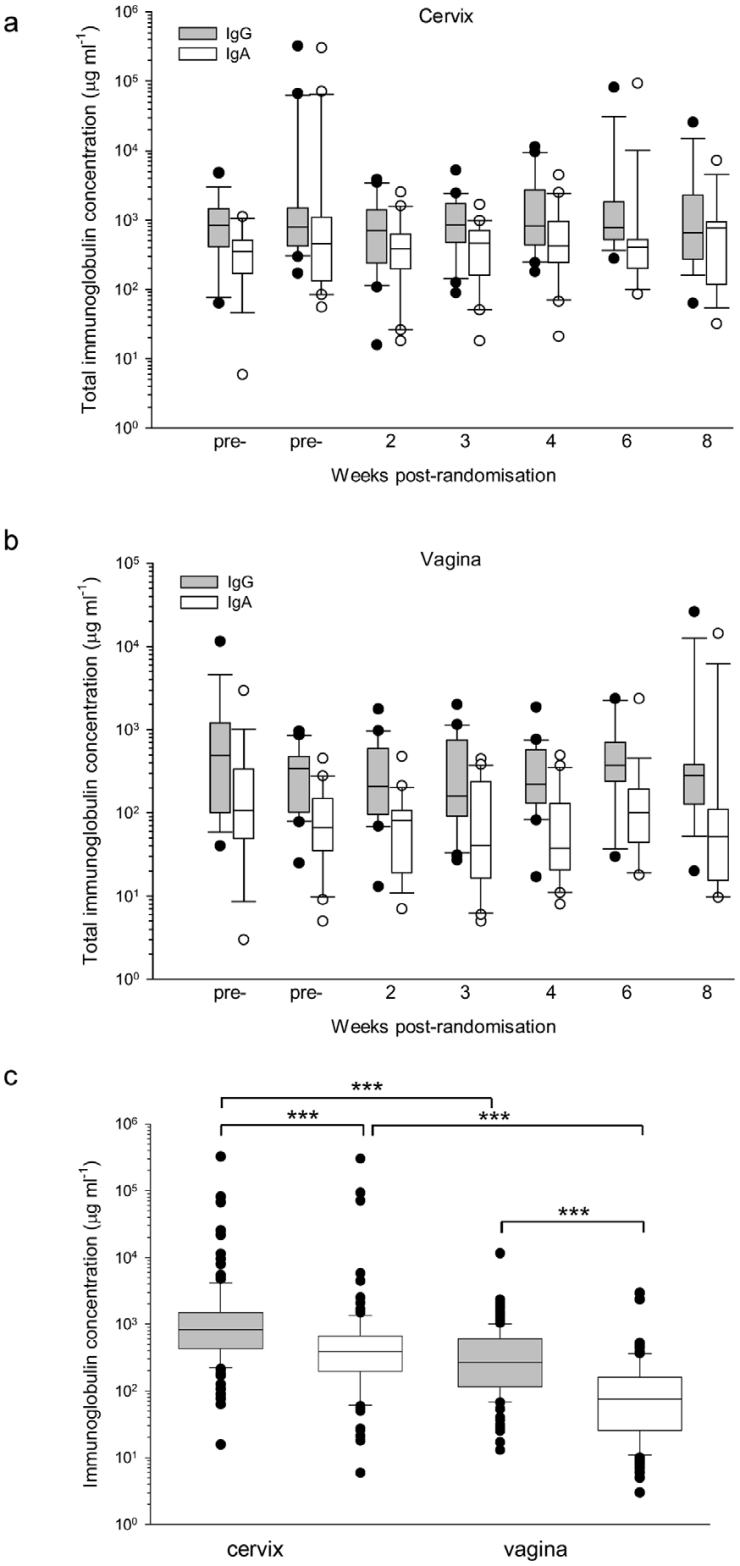
Total mucosal concentrations of IgG and IgA in trial participants. Immunoglobulin concentrations were corrected for sample volume and plotted longitudinally for cervix (a) and vagina (b). Median concentrations of neither IgG or IgA showed significant changes over time in either cervix or vagina. (c) Comparison of median concentrations showed that IgG concentrations were higher than IgA concentrations in both the cervix and vagina and that concentrations of both immunoglobulin classes was higher in the cervix compared to the vagina. These differences were statistically significantly at the ρ<0.001 level (***) (Mann Whitney ranked sum test). Box plots show 25^th^ and 75^th^ percentiles, error bars show 10^th^ and 90^th^ percentiles and median values are shown as (−).

First, total IgG and IgA content were determined in cervical and vaginal samples taken longitudinally from the trial participants. Total IgG and IgA in cervical and vaginal secretions were detected reliably and reproducibly across all visits (Figure 3a,b). Across the whole trial population, median IgG concentrations were higher than IgA concentrations in both cervical and vaginal samples (797 µg/ml versus 393 µg/ml and 267 µg/ml versus 68 µg/ml in cervix and vaginal respectively; ρ<0.001; Wilcoxon signed rank test), and both IgG and IgA levels were higher in cervical than vaginal secretions (ρ<0.001; Wilcoxon signed rank test) (Figure 3c).

Next, using the anti-gp140 ELISA, we determined whether eluates from Weck-Cel samples interfered with specific antibody detection. Pools of cervical and vaginal secretions from HIV-uninfected volunteers were spiked with HIV reference standard serum and titrated in eluted mucosal fluid. Neither cervical nor vaginal eluates had an effect on the linearity or gradient of the titration curve. Titre of IgA binding antibody was essentially unaltered and the titre of IgG was reduced by less than two-fold.

**Figure 4:**
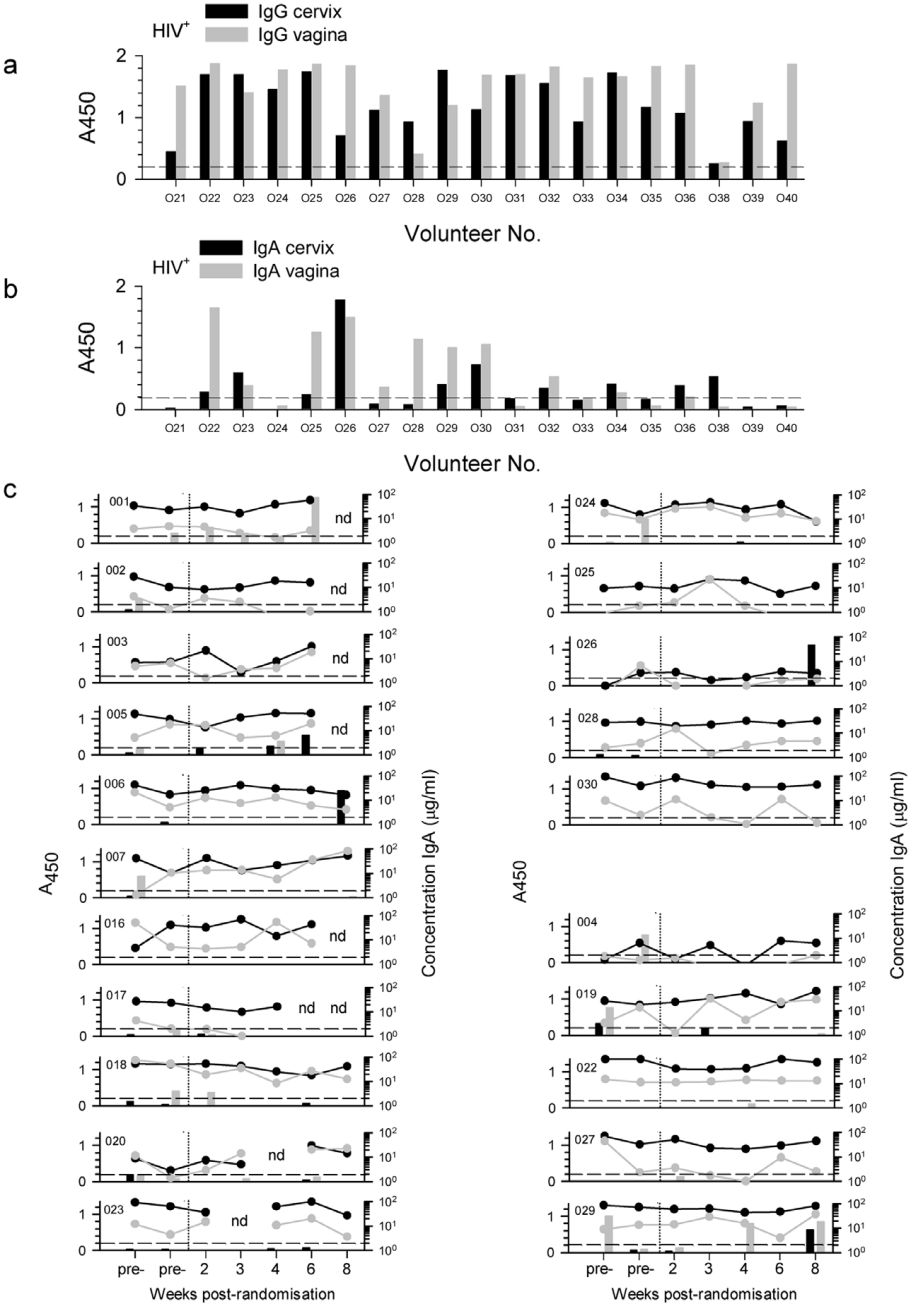
Cervical and vaginal anti-gp140 binding antibodies. Cervical and vaginal samples were screened by gp140-specific ELISA for IgG and IgA antibodies. Assay cut-offs are shown (-------) based on the analysis of secretions from 17 uninfected and unvaccinated women. Calibration results from HIV+ volunteers for IgG (a) and IgA (b) are shown for cervix and vagina. Trial participants showed no IgG gp140 binding activity (data not shown). (c) Sporadic anti-gp140 IgA binding activity (vertical histograms) was detected in cervical and vaginal samples from some vaccine trial participants (participant No. shown in top left hand corner) and reactivity was not associated with total IgA (line plots) in either cervical or vaginal samples. nd = not done.

Having established that the gp140 ELISA assays performed satisfactorily with mucosal secretions, the IgG and IgA binding antibody ELISAs were validated in an operator blinded fashion against a panel of 19 matched cervical and vaginal samples from HIV-infected volunteers and a panel of 17 matched mucosal samples from uninfected women. Antigen specific IgG antibody detection was 100% sensitive and specific in both cervical and vaginal samples (Fig. 4a). IgA binding antibody was also 100% specific and had an overall sensitivity of 79% at an A_450_ 0.1 cut-off which was reduced to 63% at the A_450_ 0.2 cut-off used in the clinical trial. In three HIV-infected subjects IgA was detected only in the cervical sample, in two subjects only in the vaginal sample and in 10 subjects IgA antibody was detected in both mucosal samples (Figure 4 b). Interestingly, vaginal samples were more likely to contain higher levels of IgG antibody, as measured by A_450_ at a dilution of 1/2, than the corresponding cervical samples and this difference was statistically significant (ρ = 0.014, Wilcoxon signed rank test); however IgA antibody levels were not statistically different (ρ = 0.465).

IgG antibodies against gp140 were detected in neither cervical nor vaginal samples from trial participants. 14/139 vaginal and 6/139 cervical samples had IgA values above the cut-off (Figure 4 c). However, in general end-point titres were low [2]–[14] with the exception of the visit 15 vaginal samples from trial participant 001 with a titre of 100; however, no corresponding reactivity was seen in the paired cervical sample. Furthermore, 9/20 reactive IgA samples were collected pre-immunisation and 7/20 were in placebo vaccine recipients, so the reactivity did not reliably indicate responses to the vaccine.

#### 2.3.3. gp140-specific IFN-γ T cell responses

**Figure 5:**
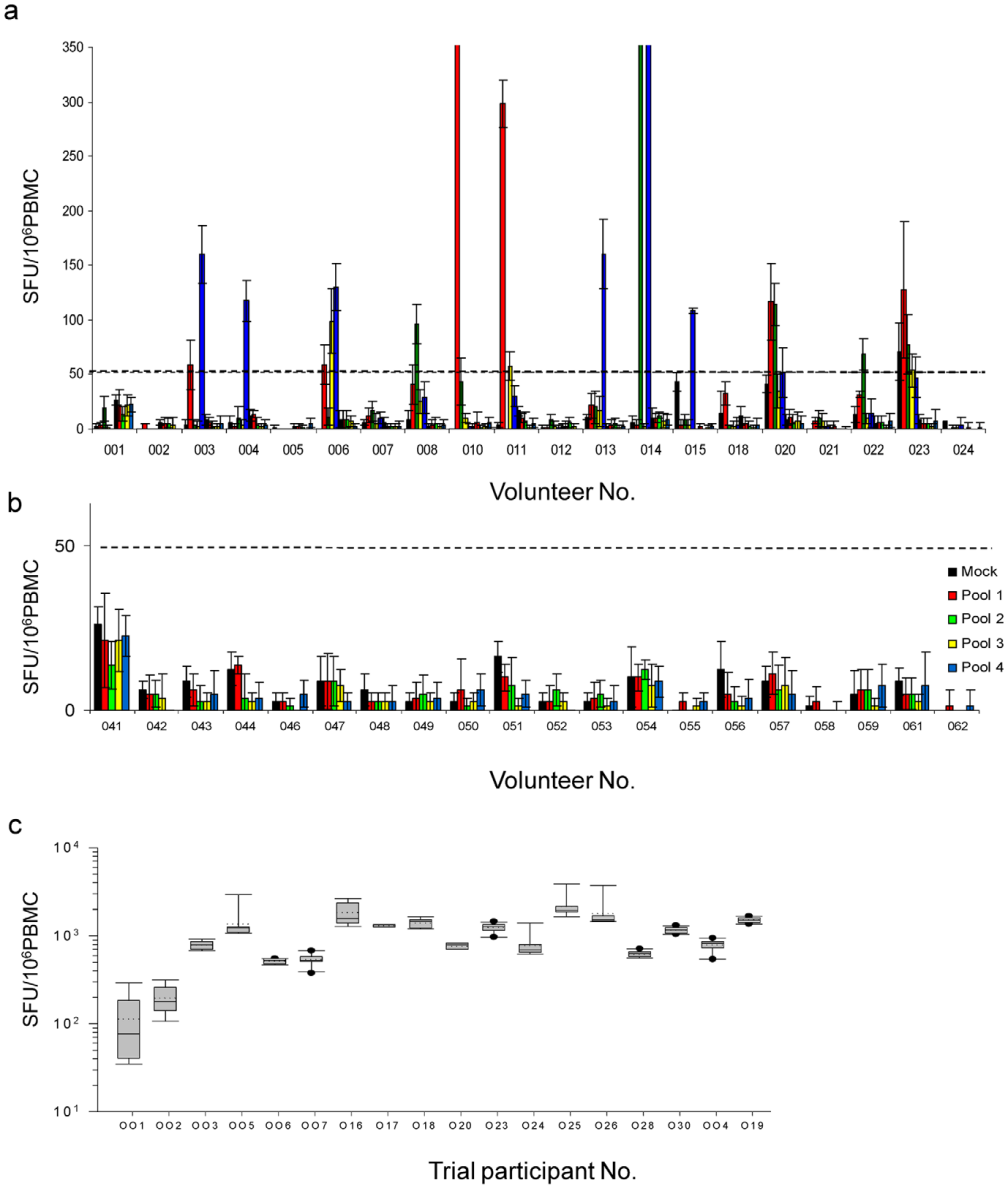
Frequency of IFN-γ secreting PBMC. (a) Calibration of the gp140-specific IFN- γ ELISpot assay using PBMC from non-clade typed HIV1-infected women. Vertical histograms show frequency of reactivity against each of four 15mer, overlapping by 11 peptide pools covering the total length of gp140. Background non-specific reactivity is also shown. (-------) indicates the cut-off frequency for positivity based upon results from PBMC taken from 20 uninfected non-vaccinated women (b). (c) With the exception of one trial participant on a single occasion (see text) no gp140-specific reactivity was detected (not shown); however all participants had FEC-specific IFN- γ secreting activity at every time point tested and the frequencies of reactive cells remained relatively consistent.

In order to calibrate the gp140-specific IFN-γ ELISpot, a panel of 20 cryopreserved PBMC from HIV-infected and 20 samples from uninfected volunteers were analysed in an operator-blinded manner for reactivity against peptide pools from a clade C gp140 sequence. Twelve of 20 samples from HIV-infected individuals reacted specifically (Figure 5a). None of the samples from uninfected individuals had specific reactivity, with background frequencies well below the assay negative cut-off of 50 SFU/10^6^ PBMC (Figure 5b). PBMC from 16 trial participants had specific reactivity to the FEC pool with a median response of 2339 SFU/10^6^ PBMC (Figure 5 c). Six of the 8 HIV-non responders had responses to FEC and the frequency of reactive cells was not significantly different to that in the HIV-responders (ρ = 0.85; Mann-Whitney sum rank test).

A total of 296 PBMC samples from trial participants were analysed. Samples from 5 participants had non-antigen background signals above the cut-off on a maximum of two occasions (range 55–132 SFU/10^6^ PBMC) and on three occasions these were in PBMC taken prior to vaccination. After background subtraction only subject 001, who had received open label gp140 vaccine, had evidence of a weak peripheral T cell response of 50 and 71.7 SFU/10^6^ PBMC against pools 2 and 4 respectively present after 6 immunisations, which was then undetectable in the subsequent menstrual cycle. All PBMC samples from trial participants tested positive for responses to FEC and responses in any one subject remained relatively stable throughout the course of the study (Figure 5c). Comparison of pre-vaccination results from the trial participants and from HIV uninfected volunteer samples used for assay calibration revealed that the median response was 788 SFU/10^6^ PBMC *i.e.* significantly lower than the median response in HIV-infected subjects (ρ = 0.022; Mann-Whitney rank sum test).

#### 2.3.4. T cells from mucosal cytobrush

**Figure 6:**
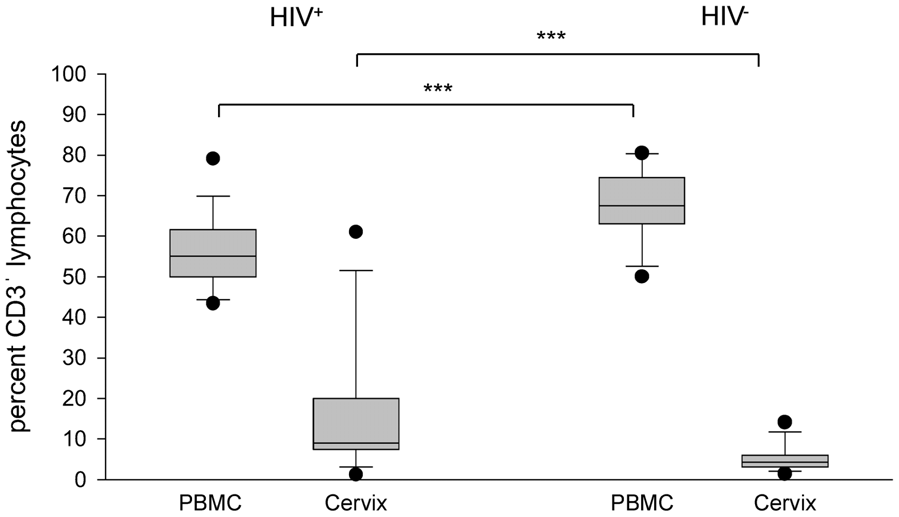
Proportion of CD3^+^ lymphocytes in cervical cytobrush samples from HIV-infected and uninfected women. For each individual the lymphocyte population was defined on PBMC by forward-side scatter profile and used to gate CD3^+^ cells recovered from the cervical cytobrush. Box plots show 25^th^ and 75^th^ percentiles, error bars show 10^th^ and 90^th^ percentiles and median values are shown as (−). Comparison of percentage CD3^+^ lymphocytes in PBMC from HIV-infected and HIV uninfected volunteers showed a statistically significant higher result in uninfected subjects (***) (ρ<0.001; Mann Whitney ranked sum test) whereas in cervical samples a higher proportion of CD3^+^ lymphocytes were measured in HIV infected volunteers (***) (ρ<0.001; Mann Whitney ranked sum test).

The feasibility of analysing T cell phenotype and function in cells recovered from cervical cytobrushing was evaluated. Paired PBMC and cervical samples were collected from 30 HIV-infected women. Lymphocyte populations were defined on PBMC samples and used to select matched cervical cells. Using, this strategy, 16 of the women had sufficient cervical T cells for further analysis. CD3^+^ cells were detected with a median frequency of 10% with a range of 1.2 to 61% which was significantly less than the proportion of CD3^+^ cells measured in PBMC (ρ<0.001; Wilcoxon signed rank test) (Figure 6). Within this CD3^+^ population the CD8^+^ phenotype predominated (median 38%); whereas CD4^+^ lymphocytes had a median frequency of 27%. The proportion of CD4^+^ T cells was significantly lower in cervical samples compared to that in fractionated PBMC (ρ = 0.009; Wilcoxon signed rank test). However, analysis of samples taken from HIV-uninfected individuals at a single time point revealed that the majority of cytobrush samples had insufficient T cells for meaningful analysis.

Longitudinal samples were taken from the vaccine trial participants; however, many of these proved unsuitable for analysis. Nonetheless, results were obtained from 5 subjects on one occasion and from 4 subjects on 2 occasions. CD3^+^ cells were detected with a median frequency of 4.3% and a range of 1.4 to 14.1% this being a significantly smaller proportion than in cytobrush samples from HIV-infected women (ρ<0.001; Mann-Whitney rank sum test) (Figure 6). The proportion of CD4^+^ T cells was usually higher than CD8^+^ cells in these samples (data not shown). However, subject 002 had 87% CD8^+^ T cells 2 weeks post-immunisations, and subject 025 had 69% CD8^+^ T cells 6 weeks post-immunisations but 31% 3 weeks post-immunisations and therefore this is unlikely to be vaccine related.

## Discussion

We conducted a novel randomised controlled trial of repeated vaginal vaccination with an HIV envelope protein in healthy young women. Recent vaginal vaccine trials have used cholera vaccines including recombinant cholera toxin B (CTB), and given promising results [7]–[10]. However, CTB is known to be a potent mucosal adjuvant, and the doses administered were in the milligram range. More recently, the immunogenicity of vaginally administered recombinant hybrid soluble HIV-1 gp160 formulated with or without the cationic lipid mucosal adjuvant DC-cholesterol was assessed in a phase 1 clinical trial [15]. A single 50 µg dose of vaccine was given in each of 3 menstrual cycles at weeks 0, 4 and 8, although the timing of vaccine administration in relation to the menstrual cycle was not specified. This study also failed to detect serum or mucosal responses to the candidate vaccine. The study reported here was markedly different in providing a repeated exposure to gp140 antigen throughout the menstrual cycle. We had conducted initial *in vitro* experiments and shown that the gp140 antigen was efficiently eluted from the carbopol carrier gel in an aqueous environment to 100% levels over 20 minutes (data not shown). We were also encouraged by pre-clinical safety data in rabbits demonstrating that repeated vaginal delivery of gp140 without an adjuvant elicited systemic IgG and mucosal cervico-vaginal IgG & IgA responses, including anti-HIV-1 neutralising antibody responses [12], [13]. The reasons for the species differences in response to vaginal gp140 between rabbits and humans may well relate to anatomical, histological and physiological factors, as the rabbit female genital tract is quite different to the human, with much of the cervico-vaginal tract covered in columnar single-cell layer epithelium [16]. Furthermore, rabbits are induced ovulators and therefore will not replicate the menstrual cycle effects on immune responsiveness reported in women [9], [10]. In order to facilitate future studies of protective efficacy we also undertook, in parallel with this clinical trial, a similar vaginal gp140 vaccination study in cynomolgus macaques [17].

The strategy of para-clinical evaluation was adopted to allow cross validation of immunogenicity between humans and macaques using standardised assays. Interestingly, the results from the macaque study showed that a similar immunisation regime delivering nine intravaginal doses over one menstrual cycle also failed to induce any detectable humoral or cellular responses, but two additional monthly cycles of the same immunisation regime lead to a significant systemic and mucosal antibody response in 2/4 animals and primed a third animal for an anamnestic response. Furthermore, the same intravaginal regimen was shown to boost a single intramuscular immunisation of gp140 given with the AS01 adjuvant, and reciprocally that a single intramuscular immunisation was boosted by subsequent vaginal immunisation [17]. Taken together, these data suggest that intravaginal immunisation with non-adjuvanted gp140 inefficiently primes vaginal mucosal or systemic immune responses, reflective of the lower genital tract being a relatively inefficient immunological inductive site. Nevertheless the boosting of parenteral immunisation seen in macaques suggests that the same intravaginal formulation of gp140 might be able to boost vaginal and systemic responses in humans following priming by a different route of administration. Although the Carbopol used in the vaginal formulation has been shown to enhance immunogenicity when given parentally [18], we have found no evidence that it has adjuvant properties when applied mucosally in the concentration included in the gel.

Calibration and validation of immunological assays is critical for reliable interpretation of clinical trial and pre-clinical data. This is particularly challenging for mucosal samples that vary widely in volume and composition. Here we used clinical materials from HIV-infected and uninfected volunteers to standardise, optimise and validate antigen-specific assays. Antibody assays applied to sera gave robust performance. Despite the paucity of HIV-specific IgA responses reported in serum and mucosal secretions of HIV-infected individuals [19], [20] our assay was sensitive enough to detect anti-gp140 IgA antibodies in the majority of individuals tested. Moreover, data from the macaque study demonstrated that the recombinant gp140 used in the clinical trial is capable of eliciting IgA responses in serum and mucosal secretions [17]. Despite 100% specificity of the assay in validation tests it is interesting that weak anti-gp140 IgA reactivity was observed in 14/139 vaginal and 6/139 cervical samples. This reactivity was shown to be non-specific as it was observed both in some pre-immune sera and placebo participants. Furthermore this reactivity was not related to total concentration of IgA in the mucosal samples. Non-specific IgA reactivity against the HIV envelope has been previously described, although its association with HIV-exposed uninfected status remains controversial [21], [22]. In this study all trial participants were classified as low risk. Significantly, multiple exposures to gp140 at a concentration probably many times higher than that seen in infectious semen failed to boost any pre-existing non-specific reactivity to gp140. Vaccination with either the active or placebo formulation had no effect on the total IgA or IgG concentrations detected in the cervix or vagina and although variability was seen between individuals the median concentrations of IgG and IgA detected in the cervix were remarkably similar to those reported by others [23] and in cynomolgous macaques [17]. However, we found lower concentrations of total vaginal immunoglobulins, and IgG and IgA in women, and IgG in macaques than previously reported.

Analysis of IFN-γ secreting cells in PBMC from HIV-infected individuals demonstrated that the gp140-ELISpot assay did not require an exact sequence match to detect reactivity. As our cohort of HIV-infected women were not virus-clade defined, it is likely that non-responders were infected with non-clade C virus. Only one trial participant demonstrated weak and transient gp140 T cell reactivity. This was also the only subject displaying a relatively high titre anti-gp140 IgA in the cervix. However, attempts to analyse cervical T cell reactivity proved to be unsuccessful. Although recovery of cells in sufficient numbers for meaningful analysis has been achieved in HIV-infected multiparous women [24], [25] low yields have limited their usefulness [26]. Our results bear this out. Even fewer T cells could be detected in cytobrushes from healthy trial volunteers and where phenotype analysis was possible, as expected, CD4^+^ cells predominated over CD8^+^ cells. Preliminary experiments were undertaken using polyclonal expansion of cells from HIV uninfected women using CD3 and CD28 co-stimulation and a novel method using CD3 and CD137 co-stimulation in the presence of survival and anti anti-apoptotic cytokines designed to bias expansion of CD8^+^ cells. However this strategy was successful in only 4 of 16 samples tested (MM-unpublished observations). Even if the efficacy of polyclonal expansion could be improved, the rate-limiting step will remain the number of precursor cells available from cytobrush samples.

To summarise, we found no evidence for adverse safety outcomes in association with the gp140 vaginal immunisation. However, we observed frequent local AEs within both active and placebo arms. Of note, the female participants in this trial disliked both the multiple invasive (i.e. vaginal, by using a speculum) sampling procedures, and vaginal product deliveries within the clinic. We are currently evaluating new vaginal self-sampling methods, and self-administered vaginal product delivery at home, which within research studies would clearly be more acceptable [27].

In conclusion, we have shown that it is feasible to conduct a HIV vaccine phase 1 trial focussing intensely on measurement of cervico-vaginal immune responses. We found no evidence in humans that immunisation with the vaginal gp140 vaccine candidate induced local or systemic immune responses. Further studies in both macaques and humans will determine whether priming by intramuscular or intranasal routes followed by vaginal boosting with gp140 leads to enhanced local and/or systemic responses.

## Supporting Information

Checklist S1 **CONSORT checklist.**
(DOC)

Protocol S1 **Trial Protocol.**
(DOC)

## References

[1] Haase AT (2010). Targeting early infection to prevent HIV-1 mucosal transmission.. Nature.

[2] Li Q, Skinner PJ, Ha SJ, Duan L, Mattila TL (2009). Visualizing antigen-specific and infected cells in situ predicts outcomes in early viral infection.. Science.

[3] Miller CJ, Li Q, Abel K, Kim EY, Ma ZM (2005). Propagation and dissemination of infection after vaginal transmission of simian immunodeficiency virus.. J Virol.

[4] The GlaxoSmithKline vaccine HPV-007 study group (2009). Sustained efficacy and immunogenicity of the human papillomavirus (HPV)-16/18 AS04-adjuvanted vaccine: analysis of a randomised placebo-controlled trial up to 6.4 years.. Lancet.

[5] Ogra LP, Ogra SS (1973). Local antibody response to poliovaccine in the human female genital tract.. J Immunol.

[6] Klavinskis LS, Bergmeier LA, Gao L, Mitchell E, Ward RG (1996). Mucosal or targeted lymph node immunization of macaques with a particulate SIVp27 protein elicits virus-specific CTL in the genito-rectal mucosa and draining lymph nodes.. J Immunol.

[7] Wassen L, Schon K, Holmgren J, Jertborn M, Lycke N (1996). Local intravaginal vaccination of the female genital tract.. Scand J Immunol.

[8] Kozlowski PA, Cu-Uvin S, Neutra MR, Flanigan TP (1997). Comparison of the oral, rectal, and vaginal immunization routes for induction of antibodies in rectal and genital tract secretions of women.. Infec Immun.

[9] Kozlowski PA, Williams SB, Lynch RM, Flanigan TP, Patterson RR (2002). Differential induction of mucosal and systemic antibody responses in women after nasal, rectal, or vaginal immunization: influence of the menstrual cycle.. J Immunol.

[10] Johannson EL, Wassen L, Holmgren J, Jertborn M, Rudin A (2001). Nasal and vaginal vaccination have differential effects on antibody responses in vaginal and cervical secretions in humans.. Infec Immun.

[11] Wang Y, Abel K, Lantz K, Krieg AM, McChesney MB (2005). The Toll-like receptor 7 (TLR7) agonist, imiquimod, and the TLR9 agonist, CpG ODN, induce antiviral cytokines and chemokines but do not prevent vaginal transmission of simian immunodeficiency virus when applied intravaginally to rhesus macaques.. J Virol.

[12] Cranage MP, Fraser CA, Stevens Z, Huting J, Chang M (2010). Repeated vaginal administration of trimeric HIV-1 clade C gp140 induces serum and mucosal antibody responses.. Mucosal Immunol.

[13] Curran RM, Donnelly L, Morrow RJ, Fraser C, Andrews G (2009). Vaginal delivery of the recombinant HIV-1 clade-C trimeric gp140 envelope protein CN54gp140 within novel rheologically structured vehicles elicits specific immune responses.. Vaccine.

[14] Currier JR, Kuta EG, Turk E, Earhart LB, Loomis-Price L (2002). A panel of MHC class I restricted viral peptides for use as a quality control for vaccine trial ELISPOT assays.. J Immunol Methods.

[15] Pialoux G, Hocini H, Perusat S, Silberman B, Salmon-Ceron D (2008). Phase 1 study of a candidate vaccine based on recombinant HIV-1 gp160 (MN/LAI) administered by the mucosal route to HIV-seronegative volunteers: the ANRS VAC14 study.. Vaccine.

[16] Barberini F, Correr S, De Santis F, Motta PM (1991). The epithelium of the rabbit vagina: a microtopographical study by light, transmission and scanning electron microscopy.. Arch Histol Cytol.

[17] Cranage MP, Fraser CA, Cope A, McKay PF, Seaman MS (2011). Antibody responses after intravaginal immunisation with trimeric HIV-1_CN54_ clade C gp140 in Carbopol gel are augmented by systemic priming or boosting with an adjuvanted formulation.. Vaccine.

[18] Krashias G, Simon A-K, Wegmann F, Kok WL, Ho LP (2010). Potent adaptive immune responses induced against HIV-1 gp140 and influenza virus HA by a polyanionic carbomer..

[19] Raux M, Finkielsztejn L, Salmon-Ceron D, Bouchez H, Excler JL (1999). Comparison of the distribution of IgG and IgA antibodies in serum and various mucosal fluids of HIV type1-infected subjects.. AIDS Res Hum Retroviruses.

[20] Mestecky J, Moldoveanu Z, Smith PD, Hel Z, Alexander RC (2009). Mucosal immunology of the genital and gastrointestinal tracts and HIV-1 infection.. J Reprod Immunol.

[21] Mestecky J, Wright P, Lopalco L, Staats HF, Kozlowski PA (2011). Scarcity or absence of humoral immune responses in the plasma or cervicovaginal lavage fluids of heavily HIV-1-exposed but persistently seronegative women.. AIDS Res Hum Retroviruses.

[22] Tudor D, Derrien M, Diomede L, Drillet AS, Houimel M (2009). HIV-1 gp41-specific monoclonal mucosal IgAs derived from highly exposed but IgG-seronegative individuals block HIV-1 epithelial transcytosis and neutralize CD4(+) cell infection: an IgA gene and functional analysis.. Mucosal Immunology.

[23] Safaeian M, Kemp T, Falk RT, Rodriguez AC, Hildesheim A (2009). Determinants and correlation of systemic and cervical concentrations of total IgA and IgG.. Cancer Epidemiol Biomarkers Prev.

[24] Kaul R, Thottingal P, Kimani J, Kiama P, Waigwa CW (2003). Quantitative ex vivo analysis of functional virus-specific CD8 T lymphocytes in the blood and genital tract of HIV-infected women.. AIDS.

[25] Gumbi PP, Nkwanyana NN, Bere A, Burgers WA, Gray CM (2008). Impact of mucosal inflammation on cervical human immunodeficiency virus (HIV-1)-specific CD8 T-cell responses in the female genital tract during chronic HIV infection.. J Virol.

[26] Bere A, Denny L, Burgers WA, Passmore J-A (2010). Polyclonal expansion of cervical cytobrush-derived T cells to investigate HIV-specific responses in the female genital tract.. Immunology.

[27] Boskey ER, Moench TR, Hees PS, Cone RA (2003). A self-sampling method to obtain large volumes of undiluted cervicovaginal secretions.. Sex Transm Dis.

