# Supplementary material for: Phase I Randomised Clinical Trial of an HIV-1CN54, Clade C, Trimeric Envelope Vaccine Candidate Delivered Vaginally
Source: PLoS One. 2011 Sep 30;6(9):e25165. doi: 10.1371/journal.pone.0025165 (PMC3184147; doi:10.1371/journal.pone.0025165)
Supplement: Protocol S1 — Trial Protocol. (DOC) [file pone.0025165.s002.doc]

**CLINICAL STUDY PROTOCOL**

| Phase I clinical trial in healthy female volunteers of reactogenicity and immunogenicity of nine vaginal immunisations with HIV CN54gp140 glycoprotein. |
| --- |

Study Identification: SG06RS02

EUDRACT NUMBER: 2007-000781-20

REC number: 07/q0803/29

Version number: 7.0

Date: 19 February 2008

sHORT tITLE: Reactogenicity and immunogenicity of vaginal CN54gp140

This study will be conducted in accordance with ICH GCP Guidelines (Directive CPMP/ICH/135/95) and the Declaration of Helsinki (1964) and subsequent amendments

CHIEF INVESTIGATOR: Prof. David JM Lewis, MD, MSc, FRCP, DTM&H

St. George’s Vaccine Institute

Centre for Infection

St. George’s - University of London

Cranmer Terrace

London SW17 0RE

Email: [sgf300@sgul.ac.uk](mailto:sgf300@sgul.ac.uk)

Tel: 00 44 20 87 25 58 26/7/8/9

Fax: 00 44 20 87 25 34 87

PRINCIPAL INVESTIGATOR

St George’s site: Prof. David JM Lewis, MD, MSc, FRCP, DTM&H

PRINCIPAL INVESTIGATOR

York Hospital site: Prof. Charles JN Lacey, MD, FRCP

York Hospital

Department of GU Medicine

31 Monkgate

York YO31 7WA

Email: [Charles.Lacey@hyms.ac.uk](mailto:Charles.Lacey@hyms.ac.uk)

Tel: 00 44 1904 725423

Fax: 00 44 1904 642116

CO-INVESTIGATORS Prof. Robin Shattock

Centre for Infection

St. George’s - University of London

Cranmer Terrace

London SW17 ORE

email: shattock@sgul.ac.uk

CLINICAL LABORATORY: The Doctors Laboratory (SGUL site)

60 Whitfield Street

London

W1T 4EU

Abraham Roodt, Clinical Trials and Data Manager

Pathology Department (York site)

York Hospital

Wigginton Road,

York, Y031 8HE

SIGNATURES (1)

PROTOCOL APPROVED BY

| Prof. David J. M. Lewis, St. George’s University of London |
| --- |
| SIGN__________________________________________ DATE_______________________ |

**I AGREE TO CONDUCT THE STUDY IN ACCORDANCE WITH THE INFORMATION CONTAINED IN THIS STUDY PROTOCOL:**

| Principal Investigator, St. George’s University of London |
| --- |
| Prof. David J.M. Lewis |
| SIGN__________________________________________ DATE_______________________  PRINT NAME: __________________________________________ |

| Principal Investigator, Department of GU Medicine, York |
| --- |
| Dr. Charles J. N. Lacey |
| SIGN__________________________________________ DATE_______________________  PRINT NAME: __________________________________________ |

PROTOCOL SYNOPSIS

| **STUDY TITLE:** | Phase 1 clinical trial in healthy female volunteers of reactogenicity and immunogenicity of nine vaginal immunisations with CN54gp140 glycoprotein. |
| --- | --- |
| **Short Title** | Reactogenicity and immunogenicity of vaginal CN54gp140 |
| **SPONSOR:** | St. George’s - University of London. |
| **CHIEF INVESTIGATOR** | Prof. David JM Lewis, MD, MSc, FRCP, DTM&H  St. George’s Vaccine Institute  Centre for Infection  St. George’s - University of London  Cranmer Terrace  London SW17 0RE |
| **PRINCIPAL INVESTIGATOR SGUL:** | Prof. David JM Lewis |
| **PRINCIPAL INVESTIGATOR York:** | Prof. Charles J N Lacey, MD, FRCP  Department of GU Medicine  31 Monkgate  York, YO31 7WA |
| **PLANNED STUDY DATES:** | April 2007-September 2008 |
| **PHASE:** | I |

| **OBJECTIVES:** | Primary objective   1. To determine the local and systemic safety of vaginal immunisations with CN54gp140 glycoprotein administered 9 times over a 3 week period.   Exploratory objectives   1. To determine the frequency of subjects mounting a cervico-vaginal IgA and IgG response to gp140 after a cycle of 9 vaginal immunisations. 2. To determine the frequency of subjects mounting a serum IgG, and IgA response to gp140 after a cycle of 9 vaginal immunisations. 3. To determine the frequency of subjects a T-cell response to gp140 in blood after a cycle of 9 vaginal immunisations. 4. To determine the frequency of cellular responses to gp140 in cervical cells after a cycle of 9 vaginal immunisations. |
| --- | --- |
| **STUDY DESIGN:** | This study will be a Phase I, randomised, two-centre, double-blind, hypothesis-generating study.  Initially one subject will be recruited and allocated to receive 9 applications of 100µg of gp140 in 3.0 mL gel (total gp140 dose 0.9 mg) in an open-label, non-randomised manner.  Following satisfactory safety review at visit 6 (after three applications) the remaining 29 subjects will be recruited as one cohort and allocated to the following groups in a double‑blind, randomised manner:  Group 1: (19 subjects) receiving 9 applications of:  100µg of gp140 in 3.0 mL gel (total gp140 dose 0.9 mg)  Group 2: Control (10 subjects) receiving 9 applications of:  3.0 mL placebo gel. |
| **SAMPLE SIZE:** | Total: 30 subjects in all. |
| **STUDY POPULATION:** | Healthy female volunteers aged 18 to 45 years. |
| **TEST PRODUCT:** | CN54gp140 glycoprotein 100µg in gel 3.0 mL (9 applications) |
| **COMPARATOR:** | 3.0 mL placebo gel (9 applications). |
| **ROUTE:** | Intravaginal |
| **DURATION OF TREATMENT:** | - The entire duration of the study (inclusive of 2 pre-study screening visits) will be approximately 15 weeks. During this period subjects will make a total of 16 visits to the clinical sites. - Each visit will be performed on an outpatient basis and there will be no overnight stays. |

| **SAFETY VARIABLES:** | 1. Symptoms (vaginal discharge, fever etc) recorded in diary card after each immunisation. Diary cards will be reviewed during visits 3, 6, 9, 12 and 15. Subjects will be able to make free text additions to the diary thereafter. 2. Physical examination to include vaginal examination. 3. Colposcopy to be done and photographically documented on screening and final visits. 4. Changes in haematological and biochemical parameters from baseline. |
| --- | --- |
| **EXPLORATORY VARIABLES:** | Blood samples will be taken at visits 1, 2, 3, 6, 10, 12, 13, 14 15 and 16. Vaginal and cervical secretion samples will be obtained by Weck-cell sponges during visits 1, 2, 12, 13, 14 15 and 16.  Cervical cell samples will be taken by cytobrush from visit 2, 12, 13, 14 15 and 16.  Samples will be tested for the following exploratory variables:   1. IgA and IgG responses to HIV gp140 protein in  - vaginal secretions - cervical secretions - serum  1. T-cell responses to HIV gp140 peptides in blood. 2. Cellular responses to gp140 in cervical cells following a cycle of 9 vaginal immunisations. |
| **STATISTICAL METHODS** | No statistical null hypothesis is formulated for this phase I study. This study is hypothesis generating.  Twenty and ten subjects in vaccine and gel only groups respectively are judged to be enough to obtain good estimates of safety and preliminary immunogenicity, to be possibly used in the design of later Phase 2a studies of immunogenicity.  Running analysis of immunogenicity data will be performed as subjects complete their final visit. An unblinded Data Monitoring Committee will evaluate immunology results. |

TABLE OF CONTENTS

[1 List of Abbreviations and Definitions of Terms 11](#__RefHeading___Toc160538478)

[2 INTRODUCTION 13](#__RefHeading___Toc160538479)

[2.1 Background 13](#__RefHeading___Toc160538480)

[2.2 Review of investigational medicinal product (IMP) 13](#__RefHeading___Toc160538481)

[2.3 Rationale for the trial 14](#__RefHeading___Toc160538482)

[2.4 Rationale for the choice of dose 14](#__RefHeading___Toc160538483)

[3 OBJECTIVES 15](#__RefHeading___Toc160538484)

[3.1 Primary Objective 15](#__RefHeading___Toc160538485)

[3.2 Exploratory Immunogenicity Objectives 15](#__RefHeading___Toc160538486)

[4 variables for assessment 15](#__RefHeading___Toc160538487)

[4.1 Primary Variables: Safety and tolerability 15](#__RefHeading___Toc160538488)

[4.2 Exploratory Variables: Immunogenicity 16](#__RefHeading___Toc160538489)

[5 Study DESIGN 16](#__RefHeading___Toc160538490)

[5.1 Allocation to treatment groups 16](#__RefHeading___Toc160538491)

[6 study population 16](#__RefHeading___Toc160538492)

[6.1 Number of Subjects 16](#__RefHeading___Toc160538493)

[6.2 Inclusion Criteria 17](#__RefHeading___Toc160538494)

[6.3 Exclusion Criteria 17](#__RefHeading___Toc160538495)

[6.4 Withdrawal of Subjects from the Study 18](#__RefHeading___Toc160538496)

[6.4.1 Interruption of dosing 18](#__RefHeading___Toc160538497)

[6.4.2 Discontinuation of dosing 18](#__RefHeading___Toc160538498)

[6.4.3 Withdrawal 19](#__RefHeading___Toc160538499)

[6.4.4 Policy for replacing withdrawals 19](#__RefHeading___Toc160538500)

[6.5 Criteria for Stopping Study 19](#__RefHeading___Toc160538501)

[7 STUDY MEDICATION 20](#__RefHeading___Toc160538502)

[7.1 Detail of Study Treatments 20](#__RefHeading___Toc160538503)

[7.2 Packaging and Labelling 20](#__RefHeading___Toc160538504)

[7.2.1 CN54gp140 protein 20](#__RefHeading___Toc160538505)

[7.2.2 Gel vehicle 21](#__RefHeading___Toc160538506)

[7.3 Drug Storage and Accountability 21](#__RefHeading___Toc160538507)

[7.4 Vaccine Reconstitution and Administration 22](#__RefHeading___Toc160538508)

[7.5 Blinding 22](#__RefHeading___Toc160538509)

[7.6 Randomisation 23](#__RefHeading___Toc160538510)

[7.7 Compliance 23](#__RefHeading___Toc160538511)

[7.8 Concomitant Medication 23](#__RefHeading___Toc160538512)

[8 STUDY CONDUCT 23](#__RefHeading___Toc160538513)

[8.1 Schedule of Investigation 24](#__RefHeading___Toc160538514)

[8.1.1 Screening cycle 24](#__RefHeading___Toc160538515)

[8.1.2 Immunisation cycle 24](#__RefHeading___Toc160538516)

[8.1.3 Sampling cycles 25](#__RefHeading___Toc160538517)

[8.1.4 Final visit 25](#__RefHeading___Toc160538518)

[8.1.5 Visit Windows 29](#__RefHeading___Toc160538519)

[8.2 Safety Assessments 29](#__RefHeading___Toc160538520)

[8.2.1 Clinical Assessments 29](#__RefHeading___Toc160538521)

[8.2.2 Laboratory Assessments 29](#__RefHeading___Toc160538522)

[8.2.3 Adverse Events 29](#__RefHeading___Toc160538523)

[8.3 Immunogenicity AssESSMENTS 33](#__RefHeading___Toc160538524)

[8.4 Total blood draw during the study 33](#__RefHeading___Toc160538525)

[9 STATISTICAL CONSIDERATIONS 33](#__RefHeading___Toc160538526)

[9.1 Data Management and analysis 33](#__RefHeading___Toc160538527)

[9.2 Sample Size 33](#__RefHeading___Toc160538528)

[9.3 Statistical Analysis Plan 34](#__RefHeading___Toc160538529)

[9.3.1 Experimental Methods 34](#__RefHeading___Toc160538530)

[9.3.2 Data Analysis 34](#__RefHeading___Toc160538531)

[9.3.3 Withdrawals 35](#__RefHeading___Toc160538532)

[10 Quality Assurance Procedure 35](#__RefHeading___Toc160538533)

[11 Investigator responsibilities 35](#__RefHeading___Toc160538534)

[11.1 Investigator Performance 35](#__RefHeading___Toc160538535)

[11.2 Ethical Considerations 36](#__RefHeading___Toc160538536)

[11.2.1 Independent Ethics Committee 36](#__RefHeading___Toc160538537)

[11.2.2 Volunteer Informed Consent 36](#__RefHeading___Toc160538538)

[11.2.3 Ethical Conduct of the Study 37](#__RefHeading___Toc160538539)

[11.2.4 Information for Subject’s General Practitioner 37](#__RefHeading___Toc160538540)

[11.2.5 Payment to Subjects 37](#__RefHeading___Toc160538541)

[11.3 Confidentiality 37](#__RefHeading___Toc160538542)

[11.3.1 Subject Confidentiality 37](#__RefHeading___Toc160538543)

[11.4 Study Documentation 37](#__RefHeading___Toc160538544)

[11.4.1 Case Report Forms, Investigator’s Study File and Record Retention 37](#__RefHeading___Toc160538545)

[11.4.2 Source Documentation 38](#__RefHeading___Toc160538546)

[11.5 Publication 39](#__RefHeading___Toc160538547)

[12 sponsor responsibilities 39](#__RefHeading___Toc160538548)

[12.1 General 39](#__RefHeading___Toc160538549)

[12.2 No Fault Compensation and Indemnity 39](#__RefHeading___Toc160538550)

[12.3 Monitoring 39](#__RefHeading___Toc160538551)

[12.4 Confidentiality 39](#__RefHeading___Toc160538552)

[12.5 Finance 39](#__RefHeading___Toc160538553)

[12.6 Audit 40](#__RefHeading___Toc160538554)

[13 Protocol Amendments 40](#__RefHeading___Toc160538555)

[14 WARNINGS, PRECAUTIONS AND CONTRA‑INDICATIONS 40](#__RefHeading___Toc160538556)

[15 REFERENCES 41](#__RefHeading___Toc160538557)

# List of Abbreviations and Definitions of Terms

ADL Activities of Daily Life

AE Adverse event

AIDS Acquired Immunodeficiency Syndrome

ALT (SGPT) Serum glutamic pyruvic transaminase

AST (SGOT) Serum glutamic oxaloacetic transaminase

BV Bacterial Vaginosis

CD4 Cluster of Differentiation 4

CRA Clinical Research Associate

CRF Clinical Record Form/Case Report Form

CRP C Reactive Protein

CT Chlamydia trachomatis

Dec Decreased

DNA Deoxyribonucleic acid

EDTA Ethylenediaminetetraacetic acid

Env Envelope

GC Gonorrhea

GCP Good Clinical Practice

GGT Gamma-glutamyl transferase

Gp140 Glycoprotein

GUM Genito-Urinary Medicine

Hb Haemoglobin

HCT Haematocrit

HEPS Highly exposed persistently seronegative

HIV Human Immunodeficiency Virus

IAVI International AIDS Vaccine Initiative

ICH International Conference on Harmonisation

IEC Independent Ethics Committee

IgA Immunoglobulin A

IgG Immunoglobulin G

IM Intramuscular

IMP Investigational Medicinal Product

IN Intra-nasal

ITT Intention to treat

IV Intravenous

LSLV Last subject last visit

LLN Lower limit of normal

MCV Mean Corpuscular Volume

MedDRA Medical Dictionary for Regulatory Activities

Mod Moderate

MRC CTU Medical Research Council Clinical Trials Unit

NIH National Institutes of Health

OTC Over the counter

PBMC Peripheral Blood Mononuclear Cells

PCR Polymerase Chain Reaction

PP Per-protocol

RBC/HPF Red Blood Cells per high power field

Req Required

SAE Serious Adverse Event

SAP Statistical Analysis Plan

SDV Source Document Verification

SGUL St. George’s University of London

ShCG Serum human Corionic Gonadotropin

SOC System-organ Class

SOP Standard Operating Procedures

SUSAR Suspected Unexpected Serious Adverse Reaction

TV Trichomonas vaginalis

ULN Upper Limit of Normal

WBC White Blood Cell

Adverse Event: An adverse event is any untoward medical occurrence in a patient or clinical trial subject administered an investigational product that does not necessarily have to have a causal relationship with this treatment. An adverse event (AE) can, therefore be any unfavourable and unintended sign (including an abnormal laboratory finding, for example), symptom, or disease temporally associated with the use of an investigational product, whether or not considered related to the investigational product. This definition includes intercurrent illnesses or injuries and exacerbation of pre-existing conditions.

Local and systemic reactions: Selected local and systemic AEs are routinely monitored in vaccine clinical trials as indicators of vaccine reactogenicity. It is recognized that each of these events, and particularly those of a systemic nature, may under some circumstances, in any individual subject, have a cause that is unrelated to the study vaccine. However, as a matter of convenience and in accordance with common clinical practice, all such events occurring within 7 days after immunisation are herein termed “local and systemic reactions.”

Serious Adverse Event: Any adverse event that suggests a significant hazard, contraindication, side effect, or precaution. These events include any experience that is fatal or life-threatening, requires or prolongs inpatient hospitalization, is permanently disabling, leads to a congenital abnormality, requires intervention to prevent permanent impairment or damage, or is an important and significant medical event that, based upon appropriate medical judgment, may jeopardize the subject.

Sponsor: An individual, company, institution, or organization which takes responsibility for the initiation, management, and/or financing of a clinical trial.

End of Trial: the End of Trial corresponds to the last visit of the last subject undergoing the trial (LSLV, Last Subject Last Visit).

# INTRODUCTION

- 1. Background

The global AIDS epidemic continues to grow. In their December 2006 AIDS Epidemic Update, UNAIDS (The Joint United Nations Programme on HIV/AIDS) and the World Health Organisation estimate:

- 39.5 million people living with HIV;
- 4.3 million new infections in 2006 – 2.8 million in sub-Saharan Africa;
- infection rates in Eastern Europe and Central Asia have risen by more than 50% since 2004; and
- 2.9 million people died of AIDS-related illnesses in 2006.

There is no effective preventive HIV vaccine currently available. The International AIDS Vaccine Initiative (IAVI) estimates that a highly effective vaccine could prevent over 70 million infections in 15 years^1^.

Traditional vaccine development is based on the principle that immunological memory will trigger an immune response of sufficient magnitude and quality to eliminate, or render innocuous, the infectious agent upon subsequent exposure. Such a conventional approach assumes that, once infected, the host can disarm the infectious agent via the adaptive immune response. However, it is unclear whether retroviruses, and particularly lentiviruses such as HIV, can ever be eliminated once the host is infected. Hence vaccine design may have to break with tradition: immunological memory may not be sufficient to confer protection, which may require constant presence of local specific immune effectors at the portal of viral entry.

The majority of new infections with HIV-1 are acquired through sexual transmission, with women being disproportionately susceptible. Induction of durable immune responses at the viral portals of entry, such as the vaginal and cervical mucosal surfaces in women, represents a critical barrier to progress in the development of effective, protective immunity. St George’s, University of London, leads a consortium of UK academic institutions in a project aiming to overcome this barrier. The project is funded by the Bill & Melinda Gates Foundation and the Wellcome Trust under the Grand Challenges in Global Health initiative, which is focused on health solutions for the developing world.

- 1. Review of investigational medicinal product (IMP)

There is a general consensus that an effective vaccine against HIV-1 will induce neutralizing antibodies directed to the viral coat protein, Env. The IMP, CN54gp140, is a recombinant Env, derived from a C‑clade viral isolate. This HIV subtype is believed to cause more than 50% of worldwide HIV-1 infections, and is predominant in southern and eastern Africa and India^2^.

The CN54gp140 protein comprises a sequence of 673 amino acids, and has been shown to be immunogenic, raising high‑titre antibodies when given systemically in mice, and intravaginally in rabbits.

To date, nine human Phase I vaccine trials have been performed with various recombinant Env proteins. All of the recombinant Env-based vaccines tested to date in both clinical and pre-clinical trials have been shown to induce high-titre neutralising antibodies to the homologous isolate, and to be very safe and well tolerated in humans. ZM96gp140 very closely resembles these recombinant Env proteins, and is thus extremely unlikely to behave in an unexpected manner with regard to immunogenicity or safety. Its biophysical and structural characteristics are almost identical to those of a recombinant Env currently in a Phase I clinical trial at St George’s, University of London (EudraCT number 2005 005983 10).

- 1. Rationale for the trial

Parenteral routes, as used in the majority of HIV-1 vaccine trials, do not usually favour induction and/or maintenance of mucosal immune responses. However, direct local administration of protein vaccines to the mucosa of the female vaginal tract can induce immune responses at the site of immunisation^3,4^. Furthermore, protection against vaginal challenge with virulent virus is possible if sufficient neutralizing antibody is applied topically, as shown using a simian‑HIV macaque model^5^.

The protocol described herein will assess the local and systemic safety and tolerability of CN54gp140 vaccine, administered intravaginally in a regimen of nine immunisations over three weeks. We will also assess the efficacy of CN54gp140 vaccine in inducing a specific immune response.

The vaccine formulation consists of CN54gp140 presented in an aqueous gel vehicle that contains ingredients used widely in vaginal products. The gel vehicle is suitable for self‑administration. A gel formulation is preferable to a liquid formulation because it will reduce leakage from the vagina in the period following administration. Such leakage is known to be a major barrier to user acceptance of vaginally delivered products.

Women appear to be disproportionately affected by HIV and, particularly in the developing world, may not be empowered to make sexual choices. Our approach utilizes a user-enabled technology that will be easy to use and cheap to manufacture for global use.

- 1. Rationale for the choice of dose

Each immunisation will contain 100µg CN54gp140 protein, meaning that a total dose of 900 µg will be given over nine immunisations.

We selected 100 µg CN54gp140 per immunisation to harmonise with ongoing clinical trials of closely related recombinant Env proteins. In one of those trials, ongoing at St George’s, University of London (EudraCT number 2005 005983 10), repeated immunisations of 100µg protein are being given via intranasal (IN) and intramuscular (IM) routes. In trials ongoing in the US, repeated immunisations of 100µg protein are via the IM route only. Judging by the preliminary data available, all immunisations with 100µg recombinant Env in these ongoing trials have so far been safe and well tolerated.

The selection of 100 µg per immunisation in the ongoing trials described above was based on dose‑ranging studies in preclinical animal models, including rodents, rabbits and non-human primates. The small differences between recombinant Env proteins used in different trials are not expected to cause significantly altered immunogenicity, so harmonizing to 100 µg protein per immunisation will enable a preliminary evaluation of intravaginal delivery versus IN and IM routes.

Studies of highly exposed persistently seronegative (HEPS) women demonstrate that repeated vaginal exposure to HIV-1 antigens may be required to maintain resistance to infection^6^. This probably reflects the short-lived nature of many mucosal immune responses, and justifies our selection of a repeated immunisation regimen. The selection of nine immunisations over three weeks reflects our desire to administer successive immunisations within a single menstrual cycle, in order to assess the safety and tolerability of frequently repeated immunisations and maximize our chances of observing an immune response, while minimizing the degree of inconvenience to trial volunteers.

In a pivotal rabbit toxicology study, CN54gp140 vaccine was given as nine repeated intravaginal immunisations over three weeks (100 µg CN54gp140 per immunisation; 900 µg total dose), mimicking the regimen proposed in this protocol. The vaccine caused no unacceptable side effects and induced a specific antibody response – detectable in both serum and vaginal secretions up to 4 weeks after the final immunisation^7^. These results form the basis of our proposal that the current protocol will be safe and is scientifically justified.

# OBJECTIVES

## Primary Objective

To determine the local and systemic safety of vaginal immunisation with CN54gp140 glycoprotein administered 9 times over a 3 week period.

## Exploratory Immunogenicity Objectives

1. To determine the frequency of subjects mounting a cervico-vaginal IgA and IgG response to gp140 after a cycle of 9 vaginal immunisations.
2. To determine the frequency of subjects mounting a serum IgG and IgA response to gp140 after a cycle of 9 vaginal immunisations.
3. To determine the frequency of subjects with a T-cell response to gp140 in blood after a cycle of 9 vaginal immunisations.
4. To determine the frequency of cellular responses to gp140 in cervical cells after a cycle of 9 vaginal immunisations.

# variables for assessment

## Primary Variables: Safety and tolerability

The following safety variables will be graded according to Appendix 2.

- Local cervical-vaginal adverse events (epithelial disruption, erythema, bleeding)
- Clinical or laboratory adverse events confirmed at examination or on repeat testing respectively

Symptoms reported in the diary cards.

## Exploratory Variables: Immunogenicity

1. A change from baseline in vaginal secretion IgA and IgG antibody responses against HIV-1 gp140.
2. A change from baseline in cervical secretion IgA and IgG antibody responses against HIV-1 gp140.
3. A change from baseline in serum IgG, IgA antibody responses against HIV-1 gp140.
4. A change from baseline in T-cell responses in blood to HIV-1 gp140.
5. A change from baseline in the frequency of cellular responses to gp140 in cervical cells after a cycle of 9 vaginal immunisations.

Immunogenicity assays will be conducted by St. Georges University of London Immunology Core Laboratory .

# Study DESIGN

This study will be conducted according to the Standard Operating Procedures (SOPs) of the St George’s Vaccine Institute and GU Medicine, York Hospital. Thirty subjects will be included in this Phase I, double centre, study of 9 vaginal immunisations of 100µg of CN54gp140 in 3.0 mL gel, and 9 applications of 3.0 mL placebo. The study will consist of 2 pre-study screening visits, 9 immunisation visits, and 5 sampling visits over a total period of 15 weeks (refer to schedule of investigation section 8.1). The 9 immunisations will be administered over a period of 3 weeks.

## Allocation to treatment groups

Initially one subject will be recruited and allocated to receive 9 applications of 100µg of gp140 in 3.0 mL gel (total gp140 dose 0.9 mg) in an open-label, non-randomised manner.

Following satisfactory safety review at visit 6 (after three applications, see section 6.4.1) the remaining 29 subjects will be recruited as one cohort and will be allocated to one of the following dosing regimens/groups in a double‑blind, randomised manner:

Group 1: 19 subjects receiving 9 applications of 100µg of CN54gp140 in 3.0 mL gel intravaginally

Group 2: 10 subjects receiving 9 applications of 3.0 mL placebo gel intravaginally [CONTROL]

# study population

## Number of Subjects

Thirty healthy female volunteers aged 18 to 45 years.

## Inclusion Criteria

Subjects will be considered eligible to enter the study if they satisfy all of the following inclusion criteria:

1. They are adult female volunteers, 18 to 45 years of age, who have signed an informed consent form following a detailed written explanation of participation in the protocol.
2. They are volunteers who are in good health as determined by medical history, physical examination and clinical judgement.
3. They are available for the duration of the study.
4. They are women who, if capable of becoming pregnant during the study, have agreed to have a pregnancy test immediately before immunisation, and to use 2 appropriate contraception methods from their first screening visit until 1 week after their final immunisation. One of those methods must be condoms without a spermicidal agent. The other method must be one of the following: physician‑prescribed oral hormonal agents; a diaphragm without a spermicidal agent; or an intrauterine device. Progestogen-only pills and injections are not suitable due to the lack of a regular menstrual cycle.
5. They have agreed not to undertake any vaginal practices other than receptive intercourse with a male or use of sanitary tampons during menses.
6. They have not donated blood during 3 months prior to study entry and agree to not donate for 3 months after the end of their participation in the study.

## Exclusion Criteria

Subjects will be considered ineligible to enter the study if they meet any of the following exclusion criteria:

1. They have hypersensitivity to any component of the vaccine used in this study.
2. They are found to be HIV antibody or HIV proviral DNA positive at the time of initial screening.
3. They have a known or suspected history of cervico-vaginal disease, malignancy or abnormality discovered at time of screening.
4. They present in the samples obtained at the screening visit:

- a clinically significant amount of protein or haemoglobin in the urine sample, determined by urine dipstick.
- a clinically significant abnormality in the haematological or biochemical assays.
- Positive tests for Hepatitis B and/or C infection
- An abnormality on cervical smear examination

An abnormal value will be defined by the ranges quoted by The Doctors Laboratory for the Vaccine Institute site and Pathology Department, York Hospital for the York site.

1. They have a known or suspected impairment of lung, heart, liver, kidney, diseases, blood disorders or immune dysfunction.
2. They are receiving immunosuppressive therapy (including systemic steroids).
3. They are receiving any medications via vaginal route.
4. They have any acute infections (including fever greater than or equal to 38°C) or any chronic disease.
5. They present a current problem with substance abuse or with a history of substance abuse which, in the opinion of the investigator, might interfere with participation in the study.
6. They have any condition which, in the opinion of the investigator, might interfere with the evaluation of the study objectives.
7. They have received an investigational agent within 3 months prior to study entry.
8. They cannot speak fluent English, or are planning to leave the area of the study site prior to the end of the study period, or are likely not to complete the study.

## Withdrawal of Subjects from the Study

A volunteer is free to withdraw from the study at any time, for any reason without prejudice to any future medical care.

- - 1. Interruption of dosing

At visits 3, 6, 9, an assessment of the diary card, safety blood sample results, and visual inspection of the cervico-vaginal mucosa by a trained operator, will determine whether further immunisations will take place. Immunisations will be interrupted in the presence of any of the following:

1. Profuse, non-menstrual vaginal bleeding requiring transfusion, hospitalisation or bed rest for more than 24 hours for which there is no reasonable alternative explanation.
2. A grade 3 or 4 clinical or laboratory event confirmed on examination or repeat testing respectively and thought to be *probably related* to treatment
3. A grade 3 or 4 clinical or laboratory event confirmed on examination or repeat testing respectively and thought to be *possibly* related to gel, although in this case the investigator may choose not to interrupt treatment based on his clinical judgement.

Study treatment may be recommenced after interruption following an adverse event at the discretion of the clinical investigator. If the event recurs, and is grade 3 or 4, then treatment should be discontinued.

- - 1. Discontinuation of dosing

#### The investigator may decide to discontinue dosing in a subject with interrupted dosing.

Immunisation must be discontinued in participants who are pregnant (confirmed positive pregnancy test). Every effort will be made to obtain documentary evidence of the outcome of pregnancy. Wherever possible, the subject will be followed up for adverse events and have safety bloods and monitoring if appropriate, but no immunogenicity samples will be obtained.

Participants who discontinue product either by choice or following advice from clinical personnel will be referred for a clinical examination, and for collection of all parameters listed at the final visit.

- - 1. Withdrawal

Withdrawal means discontinuation of study visits and immunisations, and the reasons for this should be recorded in the CRF. Examples for study withdrawal include:

- participant desire to stop participating in the trial
- investigator concerns that continuing visits is not in the best interest of the participant

A visual assessment of the cervico-vaginal mucosa will be made at visits 1, 2, 3, 6, 9, 12, 13, 14 15 and 16 and no immunisation or samples obtained if there is a break in the mucosal integrity.

Any comments (spontaneous or elicited) or complaints made by the subject and the reason for termination, date of stopping the study medication and the total amount of study medication must be recorded in the CRF and source documents.

Withdrawn subjects who have received any study medication are required (wherever possible) to undergo the final safety visit (Visit 15) procedures. Subjects who are withdrawn due to an AE will be followed-up until the event has stabilised.

The Chief Investigator will be notified within 24 hours of the decision to withdraw an individual subject.

- - 1. Policy for replacing withdrawals

If the first subject immunised withdraws or is withdrawn before safety evaluation on visit 6, or if they fail to receive three applications of study vaccine before visit 6, then they will be replaced, and another subject allocated to receive 9 applications of CN54gp140 in an open-label manner. Further replacements as necessary will be made until one subject has received three applications of study vaccine before visit 6.

There will be no further replacement of subjects withdrawn from the study in this hypothesis generating Phase I study once randomised, double-blind allocation to study groups 1 and 2 has commenced.

The withdrawal of a subject from the study should be discussed where possible with the study monitor and reported to the Sponsor.

- 1. Criteria for Stopping Study

If the first subject recruited (or a replacement if withdrawal has occurred) has received three applications of study vaccine before visit 6, and has an unsatisfactory safety review on visit 6 as defined in section 6.4.1, such that dosing would be interrupted, then the study will be put on hold pending discussions with the main ethics committee and regulatory authority.

Several severe or serious AEs, which are probable in relation to vaccination will lead to the study being terminated at that point. The decision to stop the study rests on the Chief Investigator.

An unblinded Data Montioring Committee formed at St. George’s of experts who are not in any way involved with the safety assessment of trial subjects will review the accumulating immunogenicity results, and will advise the PIs on the value of continuing the trial. If a lack of significant immune response is observed, a decision will be made by the Chief Investigator, the Principal Investigators, and the Data Monitoring Committee regarding the premature termination of the trial.

The sponsor reserves the right to terminate the study at any time.

# STUDY MEDICATION

## Detail of Study Treatments

Twenty subjects will receive repeated immunisations with active CN54gp140 vaccine, and ten will receive matching placebo vaccine. Each subject will receive nine immunisations over the course of three weeks. Each immunisation of active vaccine will contain 100 µg of CN54gp140 glycoprotein, so subjects in the active treatment group will receive a total of 900 µg.

CN54gp140 will be supplied by Polymun Scientific, Vienna, Austria, dissolved at a concentration of 530 µg/mL, in an aqueous solution, comprising 20 mM Tris buffer and 150 mM NaCl, at pH 7.4.

Polymun will also supply matching placebo aqueous solution, comprising 20 mM Tris buffer and 150 mM NaCl, at pH 7.4.

Shortly before administration to trial subjects, CN54gp140 (or placebo) will be reconstituted in an aqueous gel vehicle, Gel#2449. Each immunisation will comprise 3 mL of reconstituted vaccine.

The gel vehicle will be supplied by Particle Sciences Incorporated, Bethlehem, US, and will comprise common pharmaceutical excipients: Carbopol 974‑P 0.924%; benzyl alcohol 1.09%; sodium hydroxide 0.176%; and purified water 97.81%.

## Packaging and Labelling

- - 1. **CN54gp140 protein**

CN54gp140 and matching placebo will be supplied in single‑dose, polypropylene vials containing 0.35 mL solution.

Each vial will be labeled as follows:

| CN54gp140/placebo solution  For reconstitution of CN54gp140/placebo intravaginal vaccine gel, following the described procedure  Trial Code: SG06RS02 Subject No:  Batch No: Exp: |  | Store at ≤–55 °C and protect from light  **For clinical trial use only NOT FOR INJECTION**  Investigators: Dr D Lewis, St George’s University of London (SGUL), UK Dr C Lacey, York Hospital, UK  Sponsor: SGUL; tel 020 8725 2316 |
| --- | --- | --- |

- - 1. Gel vehicle

Gel#2449 will be supplied in single-dose, pre‑filled 5 mL syringes containing 4 mL.

Each syringe will be labeled as follows:

| Intravaginal Gel#2449  After reconstitution using CN54gp140/placebo solution, following the described procedure, contains 111 µg CN54gp140/placebo. After reconstitution, transfer the total expendable contents into a vaginal applicator and, within 8 h of reconstitution, insert into the vagina as described in the applicator product leaflet  Trial Code: SG06RS02 Subject No: *****  Date *of Reconstitution*: *****Time *of Reconstitution*: ***** |  | Gel#2449 Base Batch No:  Gel#2449 Base Expiry:  Store at room temp and protect from light  **For clinical trial use only NOT FOR INJECTION**  Investigators: Dr D Lewis, St George’s University of London (SGUL), UK Dr C Lacey, York Hospital, UK  Sponsor: SGUL; tel 020 8725 2316 |
| --- | --- | --- |

*****These fields will be left blank, for completion at the trial sites at the time of reconstitution.

## Drug Storage and Accountability

The Investigator must ensure that all IMP and gel supplies are kept in a secure area at the following temperatures:

• CN54gp140 protein at ≤–55 °C

• Gel#2449 at room temperature

All items must be accessible only to authorized individuals.

Upon receipt of supplies, the trial staff will conduct an inventory and acknowledge receipt to the supplier.

A further record must be kept of all study medication (and other medications where applicable) used during the study. This will include the description (lot numbers and expiry dates) and quantity of study medication received at the study site and date of receipt, as well as a record of when (date application administered) and to whom (subject number) it was dispensed.

At the end of the study, study medication accountability will be checked by the trial staff and trial monitors. The Sponsor and the Investigator will retain copies of the complete log.

All supplies (used and unused) will be retained at the trial sites until the Sponsor gives instructions for their return/destruction.

## Vaccine Reconstitution and Administration

Reconstitution and administration will be done at the trial sites, according to a validated, standard procedure, summarized below. Administration must be within 8 hours of reconstitution. The date and time of administration will be recorded in the CRF.

**Summary of vaccine reconstitution and administration procedure**

1. Take the 5mL syringe containing 4 mL Gel#2449, and push the plunger to the 3.2 mL mark – squirting the extruded gel into a paper towel, and pull the plunger back to the 4 mL mark
2. Draw up the entire contents of a vial of CN54gp140 (or placebo) solution, using a 1 mL syringe and 21G needle
3. Inject 0.21 mL CN54gp140 (or placebo) solution through the tip of the 5 mL syringe and onto the surface of the gel
4. Take an empty 5 mL syringe and pull the plunger back to the 1 mL mark
5. Connect the two syringes via a three‑way stopcock, and reconstitute the vaccine by passing the mixture between syringes for 20 complete cycles at a rate of approximately one cycle per second (one cycle means complete depression of the plunger of the first syringe followed by complete depression of the plunger of the second syringe)
6. After 20 complete cycles, remove the first syringe from the stopcock, and record the subject number, dose number, and date and time of reconstitution on its label
7. Transfer the contents of the syringe into an ORTHO® vaginal applicator (Janssen‑Cilag) by pushing the tip of the syringe into the tip of the applicator, forming a good seal, and completely depressing the syringe plunger
8. Instruct the volunteer to gently insert the cylinder of the applicator well into the vagina and completely depress the plunger to self-administer the vaccine
9. Instruct the volunteer to, with the plunger still depressed, hold the applicator by the cylinder and remove it – administration is now complete.
10. Check the applicator to make sure all the contents has been administered and make a note on the CRF.

## Blinding

After the first subject has received her first 3 immunisations with CN54gp140 vaccine in a non‑randomised, open‑label manner, this study will be randomised and double-blinded for the remaining 29 subjects entered into groups 1 and 2. A placebo aqueous solution will be prepared to match CN54gp140 solution. The Sponsor will appoint an unblinded Data Monitoring Committee formed at St. George’s of experts who are not in any way involved with the safety assessment of trial subjects. This Committee will consist of Prf. Robin Shattock, Prof. Martin Cranage, Dr. Alethea Cope.

## Randomisation

The randomization list linking subject numbers to treatment will be held at Polymun Scientific. Individual subject code‑break envelopes will be provided to the trial sites, in case emergency unblinding is required.

Subjects for double‑blind randomisation will be recruited as a single cohort. Subject numbers, and corresponding IMP supplies and code‑break envelopes, will be allocated to the two trial sites in blocks of five, to be used sequentially, as follows:

- St George’s site: subject numbers 001–005, 006–010 and 011–015
- York site: subject numbers 016–020, 021–025 and 026–030

If one site enrolls subjects faster than the other, blocks of subject numbers may be transferred to the faster site.

When the first subject is ready to be immunised, Polymun will reveal a subject number randomised to active treatment. This subject number will be allocated to the first subject studied, and so this subject will be ‘open-label’. If this subject is replaced then the replacement will receive the same treatment, and the same subject number plus 100 (first replacement), plus 200 (second replacement), plus 300 (third replacement) etc.

## Compliance

All immunisations will be self-applied at the study sites. The applicators will be checked after each administration and any remaining product will be noted in the CRF.

## Concomitant Medication

Study participants cannot be receiving any other medication via intra-vaginal routes. Participants will be allowed to continue with oral contraception if this forms part of their regular appropriate contraception plan. All medication will be recorded in CRF.

# STUDY CONDUCT

The St George’s Vaccine Institute and York Hospital Department of GU Medicine will recruit the subjects and perform all procedures according to the protocol and schedule of assessments (see Section 8.1).

It is anticipated that the first subject screenings will commence in April 2007 and the last study assessment will be performed by September 2008.

Each subject's participation in the study will last for approximately 15 weeks (including screening).

Both sites will enter subjects into the study simultaneously until recruitment is complete. Each site will be allowed to recruit at their own rate, and there will be no limitation on the number of subjects each site can enter, up to a maximum of 30 subjects in the study overall – excluding replacements for the first subject treated.

## Schedule of Investigation

Subjects will be required to make a total of 16 visits to the study sites.

Immunisations will take place every on Mondays, Wednesdays and Fridays, starting on day 7 (+/-1) after onset of menses. Tables 1-3 show the schedule of assessments for subjects whose immunisations commence on a Monday, Wednesday or Friday, respectively.

The menstrual cycle will be deemed to commence on the first day of menstrual bleeding: this day is designated day 0 of the cycle.

- - 1. Screening cycle

There will be 2 screening visits about 4 and 2 weeks before immunisation.

The following information and procedures will be recorded and performed as part of the screening assessments:

- Medical history
- Gender, ethnic origin, age
- Clinical laboratory evaluations and serology investigations
- Physical examination (cardiovascular, respiratory, abdominal, peripheral nervous system).
- Vaginal and cervical visual examination followed by secretion sampling and cervical cytobrush sampling
- Serum pregnancy test
- Blood sampling for immunology
- HIV Antibodies, HIV proviral DNA, Hepatitis B and C virus serology.
- Screening for genital infections: Chlamydia trachomatis, Neisseria gonorrhoea, Treponema pallidum (Syphilis), Trichomonas vaginalis bacterial vaginosis, and vaginal Candida albicans.
- Cervical smear test
  - 1. Immunisation cycle

On the first day of the menstrual cycle (considered day 0), the subject will telephone the study site to book an appointment for the first immunisation. Immunisation will commence on Day 7 (+/- 1) after onset of menses. If Day 7 falls on a Saturday, Sunday, Tuesday or Thursday, first immunisation will take place on the nearest Monday, Wednesday or Friday to that day, which corresponds to day 6 or 8 of the menstrual cycle as shown in Table 4. Subsequent immunisations will take place on Mondays, Wednesdays and Fridays.

A schedule of visits will be provided too the volunteers according to each individual’s day of onset of menses.

Table 4: Schedule of initiation of immunisation

| Onset of Menses (Day 0) | Day of first immunisation | Corresponding day after menses |
| --- | --- | --- |
| Monday | Following Monday | 7 |
| Tuesday | Following Monday | 6 |
| Wednesday | Following Wednesday | 7 |
| Thursday | Following Wednesday | 6 |
| Friday | Following Friday | 7 |
| Saturday | Following Friday | 6 |
| Sunday | Following Monday | 8 |

- - 1. Sampling cycles

Blood sampling for immunological tests will be done during the immunisation cycle on visits 3, 6 and 9 and on all visits of the sampling cycles ie visits 12, 13, 14 15 and 16. Vaginal and cervical secretion sampling and cervical cell sampling will not take place during the immunisation cycle, only on the sampling cycle ie visits 12, 13, 14 15 and 16.

Visit 16 concludes the subject’s participation in the study.

- - 1. Final safety visit

Approximately 9 weeks after initial immunisation, the subject makes a final safety visit (visit 15) to the study site for immunology and safety evaluations.

Table 1: Schedule of assessments for immunisations commencing on a Monday (i.e. first day of menses on previous Tues, Mon or Sun):

|  | **Screening**  **Cycle** | **CYCLE 1: Immunisations** | **Cycle 2: sampling** | **Cycle 3: sampling** |
| --- | --- | --- | --- | --- |

| **Visit No** |  | **1** | **2** |  | **3** | **4** | **5** | **6** | **7** | **8** | **9** | **10** | **11** |  | **12** | **13** | **14** |  | **15** | **16** |
| --- | --- | --- | --- | --- | --- | --- | --- | --- | --- | --- | --- | --- | --- | --- | --- | --- | --- | --- | --- | --- |
| **Week * /Day of the week** |  | **w 1-2** | **w 3** | **Tue, M or Sun** | **M** | **W** | **F** | **M** | **W** | **F** | **M** | **W** | **F** |  |  |  |  |  |  |  |
| **Days after onset of menses** | **0** | **7-14** | **18-25** | **0** | **6-8** | **8-10** | **10-12** | **13-15** | **15-17** | **17-19** | **20-22** | **22-24** | **24-26** | **0** | **8-10** | **14^#^** | **21^#^** | **0** | **8-10** | **21** |
| Consent |  | X |  |  |  |  |  |  |  |  |  |  |  |  |  |  |  |  |  |  |
| First day of Menses |  |  |  | X |  |  |  |  |  |  |  |  |  | X |  |  |  | X |  |  |
| Medical history |  | X |  |  |  |  |  |  |  |  |  |  |  |  |  |  |  |  |  |  |
| Physical examination |  | X |  |  |  |  |  |  |  |  |  |  |  |  |  |  |  |  | X |  |
| Vital signs |  | X |  |  | X |  |  | X |  |  | X |  |  |  | X | X | X |  | X |  |
| Pregnancy Test Blood / Urine |  | B |  |  | U |  |  | U |  |  | U |  |  |  |  |  |  |  | B |  |
| Safety Bloods |  | X |  |  | x |  |  | x |  |  | X |  |  |  | x |  | x |  | x |  |
| Urinalysis |  | x |  |  | x |  |  | x |  |  | x |  |  |  | x |  | x |  | x |  |
| HIV Ab, HIV proviral DNA |  | X |  |  |  |  |  |  |  |  |  |  |  |  |  |  |  |  | x |  |
| HBV, HCV |  | x |  |  |  |  |  |  |  |  |  |  |  |  |  |  |  |  |  |  |
| Screening for genital infections |  | X |  |  |  |  |  |  |  |  |  |  |  |  |  |  |  |  |  |  |
| Cervical smear test |  | X |  |  |  |  |  |  |  |  |  |  |  |  |  |  |  |  |  |  |
| Vaginal and cervical exam |  | X | X |  | x |  |  | x |  |  | X |  |  |  | x | x | x |  | x |  |
| Blood For Immunology Assays |  | X | X |  | x |  |  | x |  |  | X |  |  |  | x | x | x |  | x | X |
| Telephone call for appointment | x |  |  | X |  |  |  |  |  |  |  |  |  | X |  |  |  | X |  |  |
| **Immunisation** |  |  |  |  | **x** | **x** | **x** | **x** | **x** | **x** | **x** | **x** | **x** |  |  |  |  |  |  |  |
| Vaginal and cervical secretion sampling |  | X | X |  |  |  |  |  |  |  |  |  |  |  | x | x | x |  | x | X |
| Cervical cytobrush sampling |  |  | X |  |  |  |  |  |  |  |  |  |  |  | x | x | x |  | x | X |
| Diary Card Kept ^†^ |  |  |  | X | x | x | X | x | x | X | x | x | X | x | x | x | x | X | x |  |
| Diary Card Review |  |  |  |  | x |  |  | x |  |  | x |  |  |  | x |  |  |  | x |  |

**Notes:** * Day of menstrual cycle; ^#^ ± 2 days; ^†^ Subjects keep daily diary card of symptoms, vaginal symptoms, discharge, etc before and after application of investigational products until next visit. Thereafter subjects may record free-text comments into diary. Between other visits subjects may record free-text comments into diary. Completed pages of diary cards to be removed at appropriate next visits and stored in CRFs.

Table 2: Schedule of assessments for immunisations commencing on WEDNESDAY (i.e. first day of menses on previous Thur or Wed):

|  | **Screening**  **Cycle** | **CYCLE 1: Immunisations** | **Cycle 2: sampling** | **Cycle 3: sampling** |
| --- | --- | --- | --- | --- |

| **Visit No** |  | **1** | **2** |  | **3** | **4** | **5** | **6** | **7** | **8** | **9** | **10** | **11** |  | **12** | **13** | **14** |  | **15** | **16** |
| --- | --- | --- | --- | --- | --- | --- | --- | --- | --- | --- | --- | --- | --- | --- | --- | --- | --- | --- | --- | --- |
| **Week * /Day of the week** |  | **w 1-2** | **w 3** | **Thu or Wed** | **w** | **f** | **m** | **w** | **f** | **m** | **w** | **f** | **m** |  |  |  |  |  |  |  |
| **Days after onset of menses** | **0** | **7-14** | **18-25** | **0** | **6-8** | **8-10** | **10-12** | **13-15** | **15-17** | **17-19** | **20-22** | **22-24** | **25-26** | **0** | **8-10** | **14^#^** | **21^#^** | **0** | **8-10** | **21** |
| Consent |  | X |  |  |  |  |  |  |  |  |  |  |  |  |  |  |  |  |  |  |
| First day of Menses |  |  |  | X |  |  |  |  |  |  |  |  |  | X |  |  |  | X |  |  |
| Medical history |  | X |  |  |  |  |  |  |  |  |  |  |  |  |  |  |  |  |  |  |
| Physical examination |  | X |  |  |  |  |  |  |  |  |  |  |  |  |  |  |  |  | X |  |
| Vital signs |  | X |  |  | X |  |  | X |  |  | X |  |  |  | X | X | X |  | X |  |
| Pregnancy Test Blood / Urine |  | B |  |  | U |  |  | U |  |  | U |  |  |  |  |  |  |  | B |  |
| Safety Bloods |  | X |  |  | x |  |  | x |  |  | X |  |  |  | x |  | x |  | x |  |
| Urinalysis |  | x |  |  | x |  |  | x |  |  | x |  |  |  | x |  | x |  | x |  |
| HIV Ab, HIV proviral DNA |  | X |  |  |  |  |  |  |  |  |  |  |  |  |  |  |  |  | x |  |
| HBV, HCV |  | x |  |  |  |  |  |  |  |  |  |  |  |  |  |  |  |  |  |  |
| Screening for genital infections |  | X |  |  |  |  |  |  |  |  |  |  |  |  |  |  |  |  |  |  |
| Cervical smear test |  | X |  |  |  |  |  |  |  |  |  |  |  |  |  |  |  |  |  |  |
| Vaginal and cervical exam |  | X | X |  | x |  |  | x |  |  | X |  |  |  | x | x | x |  | x |  |
| Blood For Immunology Assays |  | X | X |  | x |  |  | x |  |  | X |  |  |  | x | x | x |  | x | X |
| Telephone call for appointment | x |  |  | X |  |  |  |  |  |  |  |  |  | X |  |  |  | X |  |  |
| **Immunisation** |  |  |  |  | **x** | **x** | **x** | **x** | **x** | **x** | **x** | **x** | **x** |  |  |  |  |  |  |  |
| Vaginal and cervical secretion sampling |  | X | X |  |  |  |  |  |  |  |  |  |  |  | x | x | x |  | x | X |
| Cervical cytobrush sampling |  |  | X |  |  |  |  |  |  |  |  |  |  |  | x | x | x |  | x | X |
| Diary Card Kept ^†^ |  |  |  | X | x | x | X | x | x | X | x | x | X | x | x | x | x | X | x |  |
| Diary Card Review |  |  |  |  | x |  |  | x |  |  | x |  |  |  | x |  |  |  | x |  |

**Notes:** * Day of menstrual cycle; ^#^ ± 2 days; ^†^ Subjects keep daily diary card of symptoms, vaginal symptoms, discharge, etc before and after application of investigational products until next visit. Thereafter subjects may record free-text comments into diary. Between other visits subjects may record free-text comments into diary. Completed pages of diary cards to be removed at appropriate next visits and stored in CRFs.

Table 3: Schedule of assessments for immunisations commencing on a FRIDAY (i.e. first day of menses on previous Saturday or Friday):

|  | **Screening**  **Cycle** | **CYCLE 1: Immunisations** | **Cycle 2: sampling** | **Cycle 3: sampling** |
| --- | --- | --- | --- | --- |

| **Visit No** |  | **1** | **2** |  | **3** | **4** | **5** | **6** | **7** | **8** | **9** | **10** | **11** |  | **12** | **13** | **14** |  | **15** | **16** |
| --- | --- | --- | --- | --- | --- | --- | --- | --- | --- | --- | --- | --- | --- | --- | --- | --- | --- | --- | --- | --- |
| **Week * /Day of the week** |  | **w 1-2** | **w 3** | **Sat or Fri** | **F** | **M** | **W** | **F** | **M** | **W** | **F** | **M** | **W** |  |  |  |  |  |  |  |
| **Days after onset of menses** | **0** | **7-14** | **18-25** | **0** | **6-8** | **8-10** | **10-12** | **13-15** | **15-17** | **17-19** | **20-22** | **22-24** | **25-26** | **0** | **8-10** | **14^#^** | **21^#^** | **0** | **8-10** | **21** |
| Consent |  | X |  |  |  |  |  |  |  |  |  |  |  |  |  |  |  |  |  |  |
| First day of Menses |  |  |  | X |  |  |  |  |  |  |  |  |  | X |  |  |  | X |  |  |
| Medical history |  | X |  |  |  |  |  |  |  |  |  |  |  |  |  |  |  |  |  |  |
| Physical examination |  | X |  |  |  |  |  |  |  |  |  |  |  |  |  |  |  |  | X |  |
| Vital signs |  | X |  |  | X |  |  | X |  |  | X |  |  |  | X | X | X |  | X |  |
| Pregnancy Test Blood / Urine |  | B |  |  | U |  |  | U |  |  | U |  |  |  |  |  |  |  | B |  |
| Safety Bloods |  | X |  |  | x |  |  | x |  |  | X |  |  |  | x |  | x |  | x |  |
| Urinalysis |  | x |  |  | x |  |  | x |  |  | x |  |  |  | x |  | x |  | x |  |
| HIV Ab, HIV proviral DNA |  | X |  |  |  |  |  |  |  |  |  |  |  |  |  |  |  |  | x |  |
| HBV, HCV |  | x |  |  |  |  |  |  |  |  |  |  |  |  |  |  |  |  |  |  |
| Screening for genital infections |  | X |  |  |  |  |  |  |  |  |  |  |  |  |  |  |  |  |  |  |
| Cervical smear test |  | X |  |  |  |  |  |  |  |  |  |  |  |  |  |  |  |  |  |  |
| Vaginal and cervical exam |  | X | X |  | x |  |  | x |  |  | X |  |  |  | x | x | x |  | x |  |
| Blood For Immunology Assays |  | X | X |  | x |  |  | x |  |  | X |  |  |  | x | x | x |  | x | X |
| Telephone call for appointment | x |  |  | X |  |  |  |  |  |  |  |  |  | X |  |  |  | X |  |  |
| **Immunisation** |  |  |  |  | **x** | **x** | **x** | **x** | **x** | **x** | **x** | **x** | **x** |  |  |  |  |  |  |  |
| Vaginal and cervical secretion sampling |  | X | X |  |  |  |  |  |  |  |  |  |  |  | x | x | x |  | x | X |
| Cervical cytobrush sampling |  |  | X |  |  |  |  |  |  |  |  |  |  |  | x | x | x |  | x | X |
| Diary Card Kept ^†^ |  |  |  | X | x | x | X | x | x | X | x | x | X | x | x | x | x | X | x |  |
| Diary Card Review |  |  |  |  | x |  |  | x |  |  | x |  |  |  | x |  |  |  | x |  |

**Notes:** * Day of menstrual cycle; ^#^ ± 2 days; ^†^ Subjects keep daily diary card of symptoms, vaginal symptoms, discharge, etc before and after application of investigational products until next visit. Thereafter subjects may record free-text comments into diary. Between other visits subjects may record free-text comments into diary. Completed pages of diary cards to be removed at appropriate next visits and stored in CRFs.

- - 1. Visit Windows

All visits may be made within 2 days either side of the schedule date without a protocol violation.

- 1. Safety Assessments
     1. Clinical Assessments

Each subject's medical history (including past and present illnesses, current medications taken, family medical history) will be formally assessed and recorded at screening. Volunteers’ age, gender, height, weight and ethnic origin will be recorded.

Full physical examination will be conducted at screening and at the final safety visit (Visit 15).

Weekly assessments of vital signs, diary card, safety blood sample results and a visual inspection of the cervico-vaginal mucosa by a trained operator will determine whether the following immunisations will take place, as described in Section 6.4. Any new or significant worsening of a pre-application condition will be recorded as an AE (Appendix 2).

- - 1. Laboratory Assessments

Blood samples will be taken during all sampling cycle visits and weekly during immunisation cycle (tests performed are listed on Appendix 2). All sample tubes will have labels that will specify the study code, subject initials, subject’s date of birth and sampling date.

For haematology, 4 mL of blood will be collected into EDTA tubes, inverted gently several times.

In screening and final safety visits 5 mL blood will be drawn to include microbiology assays and serum pregnancy test (ShCG). In all other visits where a blood sample will be taken, 3.5 ml of blood will be collected into plain tubes with gel, inverted gently several times for biochemistry.

HIV serology and DNA detection will be repeated at the final safety visit to detect subjects who may have contracted HIV infection during the study period, and who may therefore have developed antibody and T-cell responses due to the HIV infection that would interfere with immunogenicity variables.

- - - - 1. Urine tests

Urine will be collected on visit 1, 12, 14 and 15 for urinalysis only and on visits 3, 6, and 9 for urinalysis and pregnancy tests.

- - 1. Adverse Events
       1. Adverse Event (AE) Definition

An adverse event (AE) is defined as any untoward medical occurrence in a patient or clinical investigation subject administered an investigational product at any application that does not necessarily have to have a causal relationship with this treatment. An adverse event can, therefore be any unfavourable and unintended sign (including an abnormal laboratory finding, for example), symptom, or disease temporally associated with the use of an investigational product, whether or not considered related to the investigational product. This definition includes intercurrent illnesses or injuries and exacerbation of pre-existing conditions.

Adverse events will be assessed during each study visit, in addition to spontaneous reporting.

Subjects will be given advice by the Principal Investigators on appropriate further investigations or management if any abnormalities or complications are noted.

- - - 1. Serious Adverse Event (SAE) Definition

A serious adverse event is defined as any untoward medical occurrence that at any application:

- Results in death
- Is life-threatening (i.e., the subject was, in the opinion of the Investigator, at immediate risk of death from the event as it occurred); it does not refer to an event which hypothetically might have caused death if it were more severe.
- Requires or prolongs inpatient hospitalisation
- Results in persistent or significant disability/incapacity (i.e., the event causes a substantial disruption of a person’s ability to conduct normal life functions)
- Results in a congenital anomaly/birth defect
- Requires intervention to prevent permanent impairment or damage
- Is an important and significant medical event that may not be immediately life threatening or resulting in death or hospitalisation but, based upon appropriate medical judgment, may jeopardize the subject or may require intervention to prevent one of the other outcomes listed above.

Adverse events that do not fall into these categories are defined as **non-serious**.

It should be noted that a severe adverse event need not be serious in nature and that a serious adverse event need not, by definition, be severe.

- - - 1. Local and systemic reactions

Local and systemic reactions are defined as any of the adverse events of local or systemic nature occurring within 7 days after immunisation. These may include:

- Fever ≥ 38°C
- Rash
- Pelvic pain
- Dyspareunia
- Vulval pruritus
- Vulval erythema
- Vulval swelling
- Vaginal bleeding unrelated to menses
- Vaginal discharge
- Dysuria
  - - 1. Unexpected Adverse Reaction

No AEs are expected. Any AEes deemed related to *ZM96*gp140 would be unexpected, and therefore be classed as an unexpected adverse reaction.

- - - 1. Relationship to the Investigational Product

The relationship of the study treatment to an AE will be determined by the Investigator, based on the following definitions:

*1. Not Related*

The AE is not related if exposure to the investigational vaccine has not occurred, OR the occurrence of the AE is not reasonably related in time, OR the AE is considered unlikely to be related to use of the investigational vaccine, i.e. there are no facts (evidence) or arguments to suggest a causal relationship.

*2. Possibly Related*

The administration of the investigational vaccine and AE are considered reasonably related in time AND the AE could be explained by causes other than exposure to the investigational vaccine.

*3. Probably Related*

Exposure to the investigational vaccine and AE are reasonably related in time AND the investigational vaccine is more likely than other causes to be responsible for the AE, OR is the most likely cause of the AE.

- - - 1. Intensity (Severity)

The severity of events reported on the “Adverse Events” CRF will be determined by the Investigator as listed on tables on Appendix 1. For events not specified in the tables below the following grading should be applied:

Grade 1 (mild) No medical intervention required and/or minimal effect on daily activities such that slightly reduced for no more than 48hrs

Grade 2 (moderate) Repeated medication or medical intervention required and/or activity reduced up to half usual level for more than 48 hrs

Grade 3 (severe) Extensive/prolonged medical intervention or repeated prescribed medication; bed rest or activity reduced by >50% of usual level; can’t work

Grade 4 (extreme) Hospitalisation and/or life-threatening

- - - 1. Adverse event documentation

Each subject will be instructed to complete a diary card, to describe any symptoms experienced following the study vaccine. All adverse events and local and systemic reactions must be reported and documented. The period of observation for adverse events extends from the time the subject gives informed consent until he or she undergoes the final study examination. This may include a period before and after an active treatment of an investigational product (study vaccine) or other medication.

The Investigator will monitor all adverse events, regardless of severity, until resolution or stabilisation. All subjects experiencing adverse events - whether considered associated with the use of the study vaccine or not - must be monitored until symptoms subside and any abnormal laboratory values have returned to baseline, or until there is a satisfactory explanation for the changes observed, or until death, in which case a full pathologist's report should be supplied, if possible. All findings must be reported on an “Adverse Events” CRF and on the “Vaccine Serious Adverse Event” form, if necessary, which is part of the Investigator’s study file. All findings in subjects experiencing adverse events must be reported also in the subject's medical records.

In addition, any event resulting in a subject’s withdrawal from subsequent vaccinations or from follow-up should be reported according to the protocol instructions. All serious adverse events which occur during the course of the trial, whether considered to be associated with the study vaccination or not, have to be reported **within 24 hours** or at the latest on the following working day by telephone or fax to the Sponsor.

As far as possible, all points raised on the “Vaccine Serious Adverse Event” form need to be addressed and faxed immediately to the Monitor of the Sponsor. The event must also be documented on the “Adverse Events” CRF.

After receipt of the initial report, the Monitor will review the information and contact the Investigator if it is necessary to obtain further information for assessment of the event. Any medication or other therapeutic measures used to treat the event will be recorded on the appropriate CRF(s) in addition to the outcome of the AE.

Any urgent safety measure to protect trial subjects against any immediate hazard to their health or safety and any SUSARs must be reported to the IEC and relevant Regulatory Authorities in a timely manner. Adequate documentation will be provided to the Sponsor, showing that the IEC and relevant Regulatory Authorities have been properly notified.

If required, a follow-up report including all new information obtained on the serious adverse event must be prepared and sent to the Monitor of the Sponsor. The report should be marked "Follow-up report".

The Investigator will submit, on request, copies of all these reports to the Independent Ethics Committee and other relevant authorities.

*Post-study events*

Any adverse event occurring at any time outside the observation period or after the end of the study and considered to be caused by the study vaccine - and therefore a possible adverse drug reaction - must be reported to the Sponsor.

- 1. Immunogenicity Assessments

At pre and post immunisation visits outpatient visits according to the schedule in Table1-3, the following samples will be obtained:

- 7 mL blood taken into plain tubes with gel for collection of serum.
- 35 mL blood taken into sodium heparin tubes for separation of PBMCs according to laboratory SOPs for cellular assays.

At each time point indicated on the schedule, vaginal and cervical secretions will be taken according to a predefined SOP, and aliquoted into cryovials before immediately freezing at ultra-low temperature. Cytobrush sampling will be performed as per Tables 1-3.

Assays will be performed in batches for each volunteer once their final visit has been completed.

## Total blood draw during the study

The total blood draw during the entire study will be of 502 ml.

# STATISTICAL CONSIDERATIONS

## Data Management and analysis

Data management will be in accordance with a pre-defined Statistical Analysis Plan and data management workflow agreed between the sponsor and a subcontractor prior to any evaluation of data.

## Sample Size

A sample size of 10 is regarded as sufficient to detect immediate reactogenicity and safety in Phase 1 clinical trials. All immunogenicity read-outs are exploratory, and as this is a hypothesis-generating study, no sample size calculation is practicable, but a study group of 20 is thought to be of sufficient size.

## Statistical Analysis Plan

A full SAP will be written by a subcontractor.

- - 1. Experimental Methods
       1. Study Design

This study will be a Phase I, randomised, two-centre, double-blind, hypothesis generating study to determine the reactogenicity and immunogenicity of vaginal immunisations with 9 applications of CN54gp140.

- - - 1. Subject Populations

(a) Universal population:

All subjects enrolled and allocated a group in the study. This population will be used for analysis of demographics and all subject listings.

(b) Safety population:

All subjects who receive a vaccination and with some follow-up safety data will be included for safety analysis. This population will be used for analysis of local and systemic adverse events.

(c) Intention to treat (ITT) population:

All subjects who receive a vaccination and who provide evaluable data before and after vaccination.

(d) Per-protocol (PP) population:

All subjects who receive a vaccination and who provide evaluable data before and after vaccination and with no major protocol violations. A "major" violation is defined as a protocol deviation that is considered to have an impact on the results of the study. The protocol deviations will be identified prior to the analysis (visit out of window, in-exclusion criteria, forbidden concomitant medications) and a clinical judgment will be necessary to classify each deviation as "major" or not.

- - 1. Data Analysis
       1. Background Characteristics

Descriptive statistics (mean, standard deviation, median, minimum and maximum) for age, height and weight at enrollment will be calculated overall and by treatment group.

Distributions of subjects by ethnic origin will be summarized overall and for each treatment group.

- - - 1. Statistical Analysis
         1. Primary Variables: Safety and Tolerability

##### Summaries presenting number of subjects reporting AEs will be prepared. The original verbatim terms used by the investigator to identify adverse events in the Case Report Forms (CRFs) will be mapped to preferred terms using MedDRA. The adverse events will then be grouped by preferred terms into frequency tables according to system-organ class (SOC). When an adverse event is reported more than once by the same subject, the maximal severity and causality will be counted. Adverse events that are considered to be possibly or probably related to study treatment will be summarized separately. Serious adverse events and adverse events leading to premature withdrawal from study will be listed. Additionally, adverse events that are unrelated to vaccine will be summarized and data listings of all adverse events will be provided. Summaries presenting number of subjects reporting local and systemic reactions will be prepared separately as a subset of all the AEs.

- - - - 1. Exploratory Variables: Immunogenicity

Individual values output from immunogenicity assays will be classified as ‘no response detected’ or ‘response detected’, according to predefined cut‑offs identified during assay validation. The results will be tabulated as appropriate. There will be no statistical analysis of exploratory variables. The Data Monitoring Committee will advise whether the overall level of immunogenicity warrants the completion of the planned recruitment or a premature termination of the study.

- - 1. Withdrawals

The number of subjects who withdraw and their reason for withdrawal will be listed and tabulated.

# Quality Assurance Procedure

The St George’s Vaccine Institute and York Hospital Department of GU Medicine will conduct all study procedures in accordance with the appropriate regulatory requirements and their Standard Operating Procedures. A clinical research organisation will be subcontracted to perform independent auditing and monitoring.

# Investigator responsibilities

The Sponsor will be responsible for pharmacovigilance, monitoring, data monitoring, organising GCP audits and managing inspections by the regulatory agencies.

## Investigator Performance

All clinical work conducted under this protocol is subject to GCP rules. This may include an inspection by the Sponsor and/or Health Authority representatives at any time. The Investigator must agree to the inspection of study-related records by the Regulatory Authority representatives.

The Investigator must adhere to the following principles in addition to any applicable local regulations. The Investigator will also ensure that all staff involved in the conduct of the study are provided with copies of the protocol and all safety information before study start and are fully familiar with their role.

## Ethical Considerations

- - 1. Independent Ethics Committee

This protocol, informed consent document, and any other written information to be provided to subjects other than general non-specific information, must have the written, dated approval of a properly constituted Independent Ethics Committee (IEC).

Any substantial amendments must be submitted to the IEC and/or the appropriate national Regulatory Authority. A favourable opinion from IEC and/or a notice of no objection from the appropriate national Regulatory Authority is required prior to their implementation, unless the amendment is to reduce immediate hazard to study subjects.

A copy of the dated, written approval of the amendments must be provided to the Sponsor.

Changes involving only logistical or administrative aspects of the study (non substantial amendments) need not be submitted to the IEC for approval.

The Sponsor should keep a record of such amendments that must be made available to the appropriate national regulatory authority and/or IEC on request.

The Principal Investigator should supply the Sponsor with:

- the names and qualifications of IEC members present at the study protocol review meeting (main Ethics committee in London, site-specific in York).
- a statement from the IEC that it is organised and operates according to ICH GCP^8^, and applicable laws and regulations.
- documentation and dates of any IEC re‑approvals/re-evaluations, and of any withdrawals or suspensions of approval.
  - 1. Volunteer Informed Consent

Prior to the commencement of the study, each subject will be provided with written information giving details of the investigational product, procedures and potential risks involved during the study. Subjects will also be instructed that they are free to obtain further information from the Principal Investigator and that they are free to withdraw their consent and to discontinue their participation in the study at any time. At the same time, the subjects will be informed about the existence of an indemnification procedure.

All subject names will be filed confidentially in the Principal Investigator's files. Subjects will be identified in documentation and throughout evaluation by the study-specific subject number allocated to them on admission. The subjects will be told that all study findings will be stored on computer and handled in the strictest confidence.

Following discussion of the study with appropriate site staff, subjects will sign the study‑specific consent form, in the presence of a relevant site staff witness, to indicate that they are freely giving their informed consent.

Ongoing volunteers at the date of the implementation of this version of the protocol will be given a new version of the Information Sheet and will be asked to sign the new consent form. From the date of their signature, they will be included in the updated protocol.

- - 1. Ethical Conduct of the Study

The study will be performed in accordance with the Declaration of Helsinki (Hong Kong 1989, Somerset West 1996), Good Clinical Practice on Medicinal Products in the European Community (111/3976/88-EN - July 1990) and the Standard Operating Procedures of the Clinical sites and contractors.

- - 1. Information for Subject’s General Practitioner

As part of the informed consent process subjects will be required to consent to the notification of the General Practitioner with whom they are registered of her intended involvement in the study prior to commencement of the study. The GP will be asked to notify the Principal Investigator if they are aware of any contraindication to her participation.

- - 1. Payment to Subjects

All volunteers will be paid expenses and compensation for their inconvenience. The amounts to be paid will follow the standard rates submitted to the local IEC.

## Confidentiality

- - 1. Subject Confidentiality

The Investigator shall reassure subjects that their confidentiality will be maintained during all audits and inspections of the study site and documentation by third parties. A unique study number assigned to each subject at the start of the study, along with their initials, will be used to identify the subject on the CRF, on all study correspondence and in the study database. The Investigator will keep an identification code list and enrolment log, which will list the full name of each subject alongside the subject number assigned and the date enrolled.

## Study Documentation

- - 1. Case Report Forms, Investigator’s Study File and Record Retention

All data will be recorded on individual CRFs designed by Constella Group Ltd. All CRFs and supporting source documentation must be completed and available to Sponsor promptly, such that the timelines of the study are adhered to. The study CRA will review and collect completed CRFs or completed pages where appropriate and return these to the Sponsor.

All CRFs must be filled out completely in waterproof black ink. Any information missing or not available must be confirmed as such and an explanation written by the Investigator (or designee) on the appropriate page where necessary. All corrections of data on the CRF must be made by placing a single line through the original incorrect data and writing the correct values next to those crossed out. Incorrect data should never be obliterated and correction fluid should never be used. Where appropriate, an explanation for the error should be given. Each correction must be initialled and dated by the person making the correction. The Investigator will sign and date the cover of each completed CRF confirming that all data was collected in accordance with the protocol and any amendments.

The Investigator shall keep a copy of the Investigator’s Site File.

Completed CRFs, source documents, the Sponsor’s trial master file, data management files and clinical study report will be securely archived for 15 years following the end of the trial.

- - 1. Source Documentation

The Investigator agrees to allow inspections of the study site and any source documentation, by clinical research and audit personnel from the Sponsor, or its representatives, or external auditors or representatives of regulatory authorities. Direct access to the subjects' medical/clinical records (if applicable to the study) is necessary to verify and corroborate the data recorded on the CRFs. This procedure is termed Source Document Verification (SDV).

The following table identifies what are source data in this study:

| **Type of Data** | Source Document |
| --- | --- |
| Informed consent | Paper |
| Relevant Medical History and Current Medical Conditions | Paper/CRF |
| Physical Examination | Paper/CRF |
| Clinical Trial History | Paper/CRF |
| Blood/Plasma Donation History | Paper/CRF |
| Demographics | Paper/CRF |
| Clinical Laboratory Reports – Haematology, Biochemistry, Virology, smear test, Syphilis, CT, GC, TV | Faxed/E-mailed Report or printed report form |
| Urinalysis, Urine pregnancy tests  Screening for Candida and BV | Paper/CRF |
| Colposcopy | Paper/CRF/paper or digital photograph |
| Time of study drug administration in the unit | CRF |
| Application administered | CRF and Drug Dispensing Form |
| Time of blood sampling | Paper/CRF |
| Time of Urine sampling | Paper/CRF |
| Drug accountability | FOLDER IN PHARMACY/Investigator’s site File |
| Adverse events | Paper/CRF/Diary card |
| Concomitant Medication | Paper/CRF |
| Protocol Deviations | CRF |
| Trial Termination details | CRF |

All subjects' data will be verified against all available source data.

## Publication

If the study is to be published, the Investigators and Sponsor will normally prepare a manuscript together.

1. sponsor responsibilities

## General

The Sponsor agrees to adhere to the ICH Guidelines on Good Clinical Practice^8^ (CPMP/ICH/135/95). The Sponsor has a legal responsibility to report fully to regulatory authorities the results of this study.

## No Fault Compensation and Indemnity

St. George’s University of London and York Hospital Department of GU Medicine will provide compensation insurance against any risk incurred by a subject as a result of participation in the study arising from the negligence of their staff. As Sponsor, St. George’s University of London will provide compensation insurance against non-negligent harm occurring in clinical sites (St. George’s Vaccine Institute and York Hospital Department of GU Medicine).

## Monitoring

The study staff may not enter any subjects into treatment prior to completion of a pre-study initiation meeting conducted by the monitors.

Monitoring during the study will be undertaken at regular intervals by suitably qualified and trained personnel in accordance with their own SOPs. The purpose of monitoring is to ensure:

- compliance with the protocol
- adherence to regulatory and GCP obligations
- proper maintenance of all study documentation
- the completeness and exactness of the data entered on the CRFs
- accurate reporting of all adverse events
- drug accountability
- close liaison with the Investigator and study staff to clarify any problems which may arise during the study.

## Confidentiality

The Sponsor will not keep any material on file referring to the study subject by their full name, other than in Source Documents at the Clinical Site. The confidentiality of the subject will be respected and maintained at all times.

## Finance

This shall be the subject of separate agreements between St. George’s University of London and it’s funders, between St. George’s University of London and University of York, and between University of York and York Hospital NHS Trust.

## Audit

The study may be audited by the Sponsor, an independent auditor or a regulatory authority, either as the study is running or up to several years later.

1. Protocol Amendments

Neither the Investigator, the Sponsor or the Study Monitor will modify or alter this protocol without first obtaining agreement from all parties. Approval of any modification by the Investigator's IRB/IEC (and/or a notice of no objection from the appropriate national regulatory body/bodies in case of substantial amendments) must be obtained before implementation, except when necessary to eliminate apparent immediate hazard to the subject or where the changes do not involve subject safety or affect the subject's rights. The party initiating a modification must confirm it in writing. St Georges University of London should submit any protocol amendments as appropriate to the national regulatory body/bodies and notify all other participating Investigators of the change(s) to the protocol.

1. WARNINGS, PRECAUTIONS AND CONTRA‑INDICATIONS

No research has been conducted in relation to over dosage, and in view of the nature of this product and the mode of administration (vaginal applications) of the drug, the likelihood of over dosage is negligible.

# REFERENCES

1. IAVI. *Estimating the global impact of an AIDS vaccine*. 2006 IAVI Policy Brief.
2. Travers SA, Clewley JP, Glynn JR, Fine PE, Crampin AC, Sibande F, Mulawa D, McInerney JO & McCormack GP. *Timing and Reconstruction of the Most Recent Common Ancestor of the Subtype C Clade of Human Immunodeficiency Virus Type 1*. 2004 J Virol. 78(19): 10501–6.
3. Kozlowski PA, Lynch RM, Patterson RR, Cu-Uvin S, Flanigan TP, Neutra MR. *Modified wick method using Weck-cell sponges for collection of human rectal secretions and analysis of mucosal HIV antibody.* 2000 J Acquir Immunodef Synd 24(4):297-309.
4. Wassen L, Schon K, Holmgren J, Jertborn M, Lycke N.Reference: *Local intravaginal vaccination of the female genital tract.* 1996 Scand J Immunol. 44(4):408-14.
5. Veazey RS, Shattock RJ, Pope M, Kirijan JC, Jones J, Hu Q, Ketas T, Marx PA, Klasse PJ, Burton DR & Moore JP. *Prevention of virus transmission to macaque monkeys by a vaginally applied monoclonal antibody to HIV-1 gp120.* 2003 Nat Med. 9(3):343-6.
6. Kaul R, Trabattoni D, Bwayo JJ, Arienti D, Zagliani A, Mwangi FM, Kariuki C, Ngugi EN, MacDonald KS, Ball TB, Clerici M, & Plummer FA. *HIV-1-specific mucosal IgA in a cohort of HIV-1-resistant Kenyan sex workers*. 1999 AIDS 13(1):23-9.
7. Huntingdon Life Sciences Ltd., 2007. Study report number VZA0001/072058, entitled CN54gp140 vaccine vaginal irritation and toxicity study in the New Zealand white rabbit for 3 weeks followed by a 4 week recovery period.
8. “Guideline for Good Clinical Practice”. ICH Harmonised Tripartite Guideline (Step 4, 1 May 1996) prepared by the International Committee on Harmonisation of Technical Requirements for Registration of Pharmaceuticals for human use.
9. OMS‑ International Nonproprietary Names for Pharmaceutical Substances. Who Drug Information 19(4), 1996. (Ref. 48499).

Appendix 1 Grading of clinical and laboratory adverse events

**Based on systems in use at the MRC CTU and NIH Division of AIDS**

**Abbreviations: ULN Upper Limit of Normal LLN LowerLimitof Normal**

R_x_ Therapy Req Required

Mod Moderate IV Intravenous

ADL Activities of Daily Living Dec Decreased

OTC Over the counter

For events not specified in the tables below the following grading should be applied:

Grade 1 (mild) No medical intervention required and/or minimal effect on daily activities such that slightly reduced for no more than 48hrs

Grade 2 (moderate) Repeated medication or medical intervention required and/or activity reduced up to half usual level for more than 48 hrs

Grade 3 (severe) Extensive/prolonged medical intervention or repeated prescribed medication; bed rest or activity reduced by >50% of usual level; can’t work

Grade 4 (extreme) Hospitalisation and/or life-threatening

| **Parameter** | **Grade 1**  **Mild** | **Grade 2**  **Moderate** | Grade 3  **Severe** | Grade 4  **EXTREME** |
| --- | --- | --- | --- | --- |
| **HAEMATOLOGY** | | | | |
| Hb | 9.5-10.5g/dL | 8.0-9.4g/dL | 7.9-6.5g/dL | <6.5g/dL |
| White Blood Count  Upper  Lower | >13.000  <3,500 | >15.000  <3,000 | >20.000  <2,000 | >30.000 or  <1.000 |
| Absolute Neutrophils | 1000-1500/mm^3^ | 750-999/mm^3^ | 500-749/mm^3^ | <500/mm^3^ |
| Platelets | 75,000-120.000/mm^3^ | 50,000-74,999/mm^3^ | 20,000-49,999/mm^3^ | <20.000/mm^3^ |
| CD4 Count | 300-400/mm^3^ | <300mm^3^ | <200/mm^3^ | <100/mm^3^ |
| **BIOCHEMISTRY** | | | | |
| Potassium  Hyperkalemia  Hypokalemia | 5.0 – 5.5 meq/L  3.2 – 3.4 meq/L | 5.6 – 6.0 meq/L  3.0 – 3.1 meq/L | 6.1 – 6.5 meq/L  2.5 – 2.9 meq/L | >6.5 meq/L  <2.5 meq/L |
| Bilirubin  Hyperbilirubinemia | >1.25 – 2.0 x ULN | >2.0 – 2.5 x ULN | >2.5 – 5 x ULN | >5 x ULN |
| Glucose  Hypoglycaemia  Hyperglycaemia  nonfasting; no prior diabetes | 2.3-2.4 mmol/l  6.7-10.0 mmol/l | 2.1-2.2 mmol/l  10.1-15.0 mmol/l | 1.5-2.0 mmol/l  15.1-25.0 mmol/l | <1.5 mmol/l  >25.0 mmol/l |
| Transaminases  AST (SGOT)  ALT (SGPT)  GGT  Alk Phos | 1.25 – 2.5 x ULN  1.25 – 2.5 x ULN  1.25 – 2.5 x ULN  1.25 – 2.5 x ULN | >2.5 – 5.0 x ULN  >2.5 – 5.0 x ULN  >2.5 – 5.0 x ULN  >2.5 – 5.0 x ULN | >5.0 – 10.0 x ULN  >5.0 – 10.0 x ULN  >5.0 – 10.0 x ULN  >5.0 – 10.0 x ULN | > 10.0 x ULN  > 10.0 x ULN  > 10.0 x ULN  > 10.0 x ULN |
| Amylase | >1.0 – 1.5 x ULN | >1.5 – 2.0 x ULN | >2.0 – 5.0 x ULN | >5.0 x ULN |
| Creatinine | >1.0 – 1.5 x ULN | >1.5 – 3.0 x ULN | >3.0 – 6.0 x ULN | >6.0 x ULN |
| **URINALYSIS** | | | | |
| Proteinuria:  24 hour urine | 200 mg - 1 g loss/day  OR <0.3% OR <3 g/l | 1 – 2 g loss/day OR  0.3 – 1.0% OR 3 - 10 g/l | 2 – 3.5 g loss/day OR  >1.0% OR > 10 g/l | Nephrotic syndrome  OR >3.5 g loss/day |
| Haematuria | Microscopic only <10  RBC/HPF | >10 RBC/HPF | Gross, with or without clots OR RBC casts | Obstructive OR transfusion req |

CLINICAL PARAMETERS

| **PARAMETER** | **GRADE 1**  **MILD** | | | | **GRADE 2**  **MODERATE** | **GRADE 3**  **SEVERE** | | **GRADE 4**  **EXTREME** | | |
| --- | --- | --- | --- | --- | --- | --- | --- | --- | --- | --- |
| **CARDIOVASCULAR** | | | | | | | | | | |
| Cardiac Arrhythmia |  | | | | Asymptomatic; transient dysrhythmia, no R_x_ req | Recurrent/persistent  dysrhythmia;  symptomatic R_x_ req | | Unstable dysrhythmia,  hospitalisation and R_x_ req | | |
| Hypertension | Transient, increase >20 mm Hg diastolic BP; no R_x_req | | | | Recurrent; chronic increase >20 mm Hg diastolic BP; R_x_ req | Acute R_x_ req; outpatient | | Hospitalisation req OR end organ damage | | |
| Hypotension | Transient orthostatic hypotension with heart rate increased by >20 beats/min OR decreased by > 10 mm Hg systolic BP, no R_x_ req | | | | Symptoms OR BP decreased by >20 mm Hg systolic, correctable with oral fluid R_x_ | IV fluid req | | Mean arterial pressure <60 mm Hg, OR end organ damage, OR shock, vasopressor R_x_ req OR hospitalisation | | |
| Pericarditis | Minimal effusion | | | | Mild/mod asymptomatic effusion, no R_x_ | Symptomatic effusion, pain, EKG changes | | Tamponade OR pericardiocentesis OR surgery req | | |
| Haemorrhage, blood loss |  | | | | Mildly symptomatic, no R_x_ req | Gross blood loss OR 1-2 units transfused | | Massive blood loss OR >2 units transfused | | |
| **GASTROINTESTINAL** | | | | | | | | | | |
| Diarrhoea | Mild OR transient; 3-4 loose stools per day OR mild diarrhoea lasting <1 week | | | | Mod OR persistent; 5-10 loose stools per day OR diarrhoea lasting >1 week | >10 loose stools/day bloody diarrhoea; OR orthostatic hypotension  OR electrolyte imbalance, >2 L IV fluid req | | | Hypotensive shock OR severe electrolyte imbalance | |
| Oral Discomfort/  Dysphagia | Mild discomfort, no difficulty swallowing | | | | Difficulty swallowing but able to eat and drink | Unable to swallow solids | | | Unable to drink fluids; IV fluids req | |
| Constipation | ------------ | | | | Moderate abdominal pain 78 hours with impaction require outpatient prescription | Requiring disimpaction or hospital treatment | | | Distention with vomiting OR obstipation | |
| **PULMONARY** | | | | | | | | | | |
| Cough (for aerosol studies) | | | Transient; no R_x_ | Treatment associated cough; inhaled bronchodilator | | Uncontrolled cough; systemic R_x_ req | | | --------------- | |
| Bronchospasm Acute | | | Transient; no R_x_; FEV1 or peak flow reduced to 70% - 80% | R_x_ req; normalizes with bronchodilator; FEV1 or peak flow 50% - 69% | | No normalization with bronchodilator; FEV1 or peak flow 25% - 49%, retractions | | | Cyanosis; FEV1 or peak flow <25% OR intubated | |
| Dyspnoea | | | Dyspnoea on exertion | Dyspnoea with normal activity | | Dyspnoea at rest | | | Dyspnoea requiring O_2_ therapy | |
| **NEUROLOGICAL** | | | | | | | | | | |
| Neuro-cerebellar | | Slight incoordination  OR  Dysdiadochokinesia | | | Intention tremor OR dysmetria OR slurred speech OR nystagmus | | Ataxia requiring assistance to walk or arm incoordination interfering with ADLs | | | Unable to stand |
| Neuro-psych/mood | | --------------- | | | --------------- | | Severe mood changes requiring medical intervention; suicidal ideation | | | Acute psychosis req hospitalisation ; suicidal gesture/attempt |
| Parasthesia (burning, tingling, etc.) | | Mild discomfort; no R_x_ req | | | Mod discomfort; non-narcotic analgesia required | | Severe discomfort; OR narcotic analgesia req with symptomatic improvement | | | Incapacitating; OR not responsive to narcotic analgesia |

| **PARAMETER** | | **GRADE 1**  **MILD** | | **GRADE 2**  **MODERATE** | | **GRADE 3**  **SEVERE** | | **GRADE 4**  **EXTREME** |
| --- | --- | --- | --- | --- | --- | --- | --- | --- |
| Neuro-motor | | Mild weakness in muscle of feet but able to walk and/or mild increase or decrease in reflexes | | Mod weakness in feet (unable to walk on heels and/or toes), mild weakness in hands, still able to do most hand tasks and/or loss of previously present reflex or development of hyperreflexia and/or unable to do deep knee bends due to weakness | | Marked distal weakness (unable to dorsiflex toes or foot drop, and mod proximal weakness e.g., in hands interfering with ADLs and/or requiring assistance to walk and/or unable to rise from chair unassisted | | Confined to bed or wheel chair because of muscle weakness |
| Neuro-sensory | | Mild impairment (decreased sensation, e.g., vibratory, pinprick, hot/cold in great toes) in focal area or symmetrical distribution | | Mod impairment (mod decreased sensation, e.g., vibratory, pinprick, hot/cold to ankles) and/or joint position or mild impairment that is not symmetrical | | Severe impairment (decreased or loss of sensation to knees or wrists) or loss of sensation of at least mod degree in multiple different body sites (i.e., upper + lower extremities) | | Sensory loss involves limbs and trunk |
| Eye | |  | | Mild pain, visual changes, conjunctivae erythema, abnormal slit lamp | | Loss of vision, clinically diagnosed uveitis, mod-severe pain, glaucoma | | --------------- |
| Headache | | Mild, no R_x_ req, OR over the counter medication | Mod; OR requiring regular OTC or occasional prescription only medication | | | Severe; intractable; OR requiring repeated prescription only medication | | Requiring hospitalisation, or associated with neurological, respiratory or cardiovascular abnormalities |
| **MUSCULOSKELETAL** | | | | | | | | |
| Arthralgia/Arthritis | | Arthralgia | | Arthralgia with joint effusion or moderate impairment of activity | | Frank arthritis with or without effusion OR resulting in severe impairment of activity | | Hospitalisation |
| Myalgia | | Myalgia without limitation of activity | | Muscle tenderness at other than injection site or with moderate impairment of activity eg difficulty climbing stairs | | Frank myonecrosis OR with severe impairment of activity eg can’t climb stairs | | Hospitalisation |
| GENERAL | |  | |  | |  | |  |
| Fever  Oral>12 hours | 37.7 - 38.9°C  (100.0 – 101.5°F) | | 39.0 – 39.7°C  (101.6 – 102.9°F)  OR max temp of 103°F | | 39.8 – 40.5°C  (103 - 105°F)  OR max temp of 103.5°F | | >40.5°C (105°F)  OR max temp of >105°F | |

GENITAL REACTIONS

| PARAMETER | **Grade 1** | **Grade 2** | **Grade 3*** |
| --- | --- | --- | --- |
|  | **Mild** | **Moderate** | **Severe*** |
| **Symptoms and signs related to single application/acute genital toxicity/allergy** | Mild itching, soreness, slight redness | Moderate soreness and pain with vulval erythema and swelling | Some combination of frank erythema, pain, vulval oedema, rash/urticarial lesions spreading onto the abdomen/thighs, sloughing of mucosa |
| **Symptoms of dysuria or pruritus or dyspareunia after multiple applications** | Mild lasting <3 days  Or  Moderate lasting <24 hours | Mild lasting >3 days  Or  Moderate lasting >24 hours | Severe |
| **Signs of epithelial disruption** | Superficial, <1 swab-tip** | Superficial, >1< 4 swab-tips** | Deep or  Superficial, ≥4 swab-tips** |
| **Signs with epithelium intact, blood vessels intact** | Local/diffuse slight redness  Or  Local moderate/severe redness | Diffuse moderate/severe redness | Oedema  Or  Diffuse moderate/severe redness with Grade 2 symptoms |
| **Signs with epithelium intact, blood vessels disrupted** | Single or multiple <1 swab-tip** | Single or multiple >1 swab-tip** |  |
| **Unexpected vaginal bleeding not related to menses** | Spotting <7 days | Spotting >7 days  Fresh blood <4 days | Fresh blood >4 days  Profuse any duration |

*grade 4 is defined as extreme **swab-tip is 5 x 10m

Appendix 2 Outline clinical laboratory analysis

| ***Haematology*** | ***Biochemistry*** |
| --- | --- |
| Haemoglobin | Sodium |
| Red blood Cells | Potassium |
| HCT ratio (PCV) | Phosphate |
| MCV | Calcium |
| White blood cells | Chloride |
| Differential white cell count | Urea |
| Neutrophils | Creatinine |
| Lymphocytes | Alkaline Phosphatase |
| Monocytes | Alanine Transferase |
| Basophils | Aspartate Transferase |
| Eosinophils | Gamma GT |
| Platelets | Lactic dehydrogenase |
|  | Bilirubin |
|  | Total Protein |
|  | Albumin  C reactive protein (CRP) |
| ***Microbiology*** | |
| Hepatitis B Surface antigen***#*** |  |
| Hepatitis C antibody***#*** |  |
| HIV 1 and 2 antibody |  |
| HIV 1 DNA PCR  Syphilis, CT, GC, TV, BV, Candidosis***#*** |  |

**#** = pre-study screening, only
